# Evolution of major flowering pathway integrators in Orchidaceae

**DOI:** 10.1007/s00497-023-00482-7

**Published:** 2023-10-12

**Authors:** Yesenia Madrigal, Juan F. Alzate, Natalia Pabón-Mora

**Affiliations:** 1 Facultad de Ciencias Exactas y Naturales, Instituto de Biología, Universidad de Antioquia, Medellín, Colombia; 2 Facultad de Medicina, Centro Nacional de Secuenciación Genómica, Sede de Investigación Universitaria, Universidad de Antioquia, Medellín, Colombia

**Keywords:** Flowering, Gene evolution, Genetic regulatory network, Orchidaceae, WGD

## Abstract

The Orchidaceae is a mega-diverse plant family with ca. 29,000 species with a large variety of life forms that can colonize transitory habitats. Despite this diversity, little is known about their flowering integrators in response to specific environmental factors. During the reproductive transition in flowering plants a vegetative apical meristem (SAM) transforms into an inflorescence meristem (IM) that forms bracts and flowers. In model grasses, like rice, a flowering genetic regulatory network (FGRN) controlling reproductive transitions has been identified, but little is known in the Orchidaceae. In order to analyze the players of the FRGN in orchids, we performed comprehensive phylogenetic analyses of *CONSTANS-like/CONSTANS-like 4* (*COL/COL4), FLOWERING LOCUS D (FD), FLOWERING LOCUS C/FRUITFULL (FLC/FUL)* and *SUPRESSOR OF OVEREXPRESSION OF CONSTANS 1* (*SOC1)* gene lineages. In addition to *PEBP* and *AGL24*/SVP genes previously analyzed, here we identify an increase of orchid homologs belonging to *COL4,* and *FUL* gene lineages in comparison with other monocots, including grasses, due to orchid-specific gene lineage duplications. Contrariwise, local duplications in Orchidaceae are less frequent in the *COL*, *FD* and *SOC1* gene lineages, which points to a retention of key functions under strong purifying selection in essential signaling factors. We also identified changes in the protein sequences after such duplications, variation in the evolutionary rates of resulting paralogous clades and targeted expression of isolated homologs in different orchids. Interestingly, vernalization-response genes like *VERNALIZATION1 (VRN1)* and *FLOWERING LOCUS C (FLC)* are completely lacking in orchids, or alternatively are reduced in number, as is the case of *VERNALIZATION2/GHD7 (VRN2)*. Our findings point to non-canonical factors sensing temperature changes in orchids during reproductive transition. Expression data of key factors gathered from *Elleanthus auratiacus*, a terrestrial orchid in high Andean mountains allow us to characterize which copies are actually active during flowering. Altogether, our data lays down a comprehensive framework to assess gene function of a restricted number of homologs identified more likely playing key roles during the flowering transition, and the changes of the FGRN in neotropical orchids in comparison with temperate grasses.

**Supplementary Information:**

The online version contains supplementary material available at 10.1007/s00497-023-00482-7.

## Introduction

The floral transition resulting in the change from vegetative to reproductive phase is a critical developmental step in the angiosperms life cycle. In monocot model species like rice (*Oryza sativa*), the reproductive transition includes the meristem identity change from the vegetative shoot apical meristem (SAM) that forms leaves, to a reproductive inflorescence meristem (IM) that develops branch (BM) and spikelet (SM) meristems resulting in a compound inflorescence with floral meristems (Itoh et al. 2005; Tanaka et al. 2013). This process is regulated by endogenous and environmental factors, which merge into four main pathways: photoperiod (light response), vernalization (cold response), autonomous, and hormonal signaling (Lee & An 2015; Levy & Dean 1998; Parcy 2005). In *O. sativa*, the flowering genetic regulatory network (FGRN) includes promoters and repressors that control meristem transition, fate, and identity. In short days (SD), *Heading date 1* (*Hd1,* a *CONSTANS- CO,* homolog) is activated early on and forms a complex with the florigen *Heading date 3a* (*Hd3a*, a *FLOWERING LOCUS T-FT* homolog) that plays a critical role in mediating the photoperiod flowering signal (Kojima et al. 2002; Komiya et al. 2008; Tamaki et al. 2007; Tsuji et al. 2008). Conversely, in long days (LD), *Hd1* is converted into a transcriptional repressor (Kojima et al. 2002; Komiya et al. 2008). However, in LD rice cultivars, *RICE FLOWERING LOCUS T1 (RFT1)*, and *OsFTL4,* both *Hd3a* paralogs, are recruited for floral induction (Gu et al. 2022; Komiya et al. 2008). All the flowering signals including *Early heading date 1 (Ehd1,* a B-type response regulator)*, Hd3a* and *RFT1* are negatively regulated by the *Oryza sativa Grain number, Plant Height and Heading date 7 (Ghd7,* a *CO-*homolog*)* and *CONSTANS-LIKE4 (OsCOL4, CO-* homolog) (Choi et al. 2014; Endo-Higashi and Izawa 2011)*.* However, if *FT* signaling is in place, and has bypassed all suppressors, the rice homolog of *FLOWERING LOCUS D* (*FD*, a *bZIP* homolog) OsFD1, interacts with Hd3a via the 14-3-3 proteins to form a florigen activation complex (FAC) (Taoka et al. 2011). This FAC induces the transcription of *OsMADS14* and *OsMADS15* (the *APETALA1/FRUITFULL* homologs) in the shoot apex during floral transition (Taoka et al. 2013; Taoka et al. 2011; Tsuji et al. 2013). In parallel, *OsMADS50* and *OsMADS51* (the *SUPRESSOR OF CONSTANS 1* homologs), together with *OsMADS22* and *OsMADS47* (the *AGAMOUS Like 24/SHORT VEGETATIVE PHASE* homologs) promote floral meristem identity, and only *OsMADS55* (another *AGL24/SVP* gene) represses flowering (Fornara et al. 2008; Lee et al. 2012, 2004).

Although the flowering genetic regulatory network (FGRN) is relatively similar across grasses (Higgins et al. 2010; Leiboff and Hake 2019; Qin et al. 2017), in crown pooids like wheat (*Triticum aestivum*) and barley (*Hordeum vulgare*), there are additional vernalization responses. Flowering is determined by allelic variation at the *VERNALIZATION1* (*VRN1,* an *AP1/FUL* homolog*)* and/or *VRN2* (a *CO-like* homolog) loci (Preston and Kellogg 2008; Trevaskis et al. 2003, 2007). *VRN2* alleles can directly or indirectly repress *VRN1* alleles in LD, resulting in flowering repression (Trevaskis et al. 2007). In addition, during vernalization and/or exposure to SD, *VRN2* transcription is reduced, resulting in an up-regulation of *VRN1* and triggering flowering (Preston and Kellogg 2008; Trevaskis et al. 2003). Other genes, including *FLC (FLOWERING LOCUS C*) homologs, also repress the flowering inducers prior to cold exposure (Alexandre and Hennig 2008; Michaels and Amasino 1999; Searle et al. 2006). In barley, mutants of flowering repressors such as *hvvrn2* and *hvos,* result in plants with no cold exposure requirements and early flowering (Chen and Dubcovsky 2012; Distelfeld and Dubcovsky 2010; Greenup et al. 2010; Woods et al. 2016).

While the FGRN has been relatively well studied in grasses, less is known about the genetic mechanisms of flowering in non-model monocots, including orchids. With ca. 29,000 species, the Orchidaceae is one of the most diverse groups of ornamental angiosperms with very attractive flowers, many of which also have extensive vegetative phases in their life cycles, hindering cultivation and large-scale maintenance of flowering individuals (Hew and Yong 2004; Huang et al. 2021; Wang et al. 2017). The isolation and characterization of flowering genes, including *CO*, *FT*, *FUL*, *SOC1* and *SVP/AGL24*, have only been made in few commercial, mostly temperate orchids, like *Cymbidium*, *Dendrobium*, *Oncidium* and *Phalaenopsis* (Huang et al. 2021; Wang et al. 2017, 2019).

Assessing the homology of all genes conforming the FGRN in orchids is important for several reasons, namely: (1) the occurrence of independent whole genome duplications (WGD) across angiosperm diversification has changed the gene complements in orchids in comparison to model monocots, like grasses. (2) FGRN genes belong to different gene lineages that have different evolutionary histories in angiosperms. And finally, (3) Gene copy number and homology for all copies needs to be established prior to expression and functional characterization of the FGRN. We have previously implemented large-scale phylogenetic reconstructions for the *FT* and the *AGL24/SVP* genes across angiosperms, with a special focus in orchids. Such approach has proven valuable in narrowing down the putative genes more likely to control reproductive transitions and to assign them a hypothetical role as promoters or repressors (Madrigal et al. 2021; Ospina-Zapata et al. 2020; Ramirez-Ramirez et al. 2021). In order to understand the evolution of the FGRN in Orchidaceae, here we performed comprehensive ML analyses of the *CO/COL4, FD, FLC/FUL* and *SOC1* gene lineages in angiosperms, with a special focus on Orchidaceae. We evaluate the expression of all target genes in *Elleanthus aurantiacus,* a tropical and terrestrial member of the Orchidaceae, with biannual flowering seasons using both RT-PCR and RNA-seq. Our results allow us to propose a hypothetical FGRN for orchids with significant variations in copy number and expression patterns when compared to the canonical rice FGRN.

## Materials and Methods

### Homolog isolation of flowering candidate genes

In order to isolate *CONSTANS-like/CONSTANS-like 4* (*COL/COL4), FLOWERING LOCUS D (FD), FLOWERING LOCUS C/FRUITFULL (FLC/FUL)* and *SUPRESSOR OF OVEREXPRESSION OF CONSTANS 1* (*SOC1)* homologs, searches across major angiosperm lineages were made using BLASTN (Basic local alignment search tool) (Altschul et al. 1990) on public repositories and on our own databases. We included as queries canonical genes in the FGRN of *Arabidopsis thaliana, Oryza sativa,* and orchid homologs available from the literature or specialized databases (Supplementary Table S1). The databases searched included: NCBI (https://www.ncbi.nlm.nih.gov/genbank/), OneKP (https://db.cngb.org/onekp/), Phytozome (https://phytozome.jgi.doe.gov/pz/portal.html), the vanilla Genome hub (https://vanilla-genome-hub.cirad.fr/) Orchidbase 4.0 (http://orchidbase.itps.ncku.edu.tw/est/home2012.aspx), and Orchidstra 2.0 (http://orchidstra2.abrc.sinica.edu.tw/orchidstra2/index.php) (Carpenter et al. 2019; Chao et al. 2017; Tsai et al. 2013). Searches were also done in our own transcriptomes generated for non-model neotropical plant species which include the Magnoliid *Aristolochia fimbriata* and *Saruma henryi* (Pabón-Mora et al. 2015; Peréz-Mesa et al. 2019); Cloranthaceae members like, *Chloranthus spicatus*, *Hedyosmum goudotianum*, and *Sarcandra chloranthoides;* the eudicots: *Bocconia frutescens, Borojoa patinoi, Brunfelsia australis* and *Streptosolen jamesonii* (Arango-Ocampo et al. 2016; Ortiz-Ramírez et al. 2018; Salazar-Duque et al. 2021); and the Monocots: *Cattleya trianae, Elleanthus aurantiacus, Epidendrum frimbriatum, Gomphichis scaposa, Hypoxis decumbens, Masdevallia coccinea, Masdevallia wendlandiana, Maxillaria aurea, Miltoniopsis roezlii, Oncidium “*Gower Ramsey*”, Oncidium “*Twinkle*”, Stelis pusilla, Tolumnia “*Cherry red x Ralph yagi” *and Vanilla aphyla* (Madrigal et al. 2017; Ospina-Zapata et al. 2020; Ramirez-Ramirez et al. 2021).

### Phylogenetic analyses of flowering candidate genes

To analyze the evolution of the *COL/COL4, FD, FLC/FUL* and *SOC1* gene lineages separate matrices were generated with all isolated homologs. Sequences were cleaned manually to keep only the CDS using Aliview (Larsson 2014). Then, the sequences were aligned using the online version of the software MAFFT (mafft.cbrc.jp/alignment/software/ (Katoh et al. 2018)) with a gap opening penalty of 3.0 and offset value of 1.0. The phylogenetic hypothesis were done by maximum likelihood (ML) using the desktop version of IQ-TREE software (http://www.iqtree.org; Minh et al. 2020; Nguyen et al. 2015). The molecular evolution model that best fits to the data was found with ModelFinder on IQ-TREE (Kalyaanamoorthy et al. 2017). The branch support was calculated with Ultrafast Bootstrap (UFBS) of 1000 pseudo-replicas, also available in IQ-TREE (Hoang et al. 2018). The trees obtained were observed using FigTree v1.4.4 (http://tree.bio.ed.ac.uk/software/figtree/). To identify and confirm duplications in each gene lineage we gave special attention to genes from species with a sequenced genome (Supplementary Table S1). Specifically for orchids, we included the genomes of *Apostasia shenzhenica, Dendrobium catenatum, Phalaenopsis aphrodite, Phalaenopsis equestris, Platanthera guangdongensis, Platanthera zijinensis* and *Vanilla planifolia.* In all gene trees genomic information was essential to pointing out large-scale duplication events and/or intra-specific duplication events. The latter category remains to be confirmed as the data derived from transcriptomic analyses fails to distinguish intraspecific duplicates from splicing variants, but in most cases variation among sequences is larger than 5%, and not due to indels, suggesting these sequences are most likely copies. Nevertheless, genome sequencing for mon-model orchids will eventually confirm our inferences.

### Relative rates of evolution

To test for changes in the selection constraints in the duplicate gene lineages found, we performed a series of Likelihood Ratio Tests (LRTs) using the branch-specific model implemented by the CodeML in the PAML package v.4.8 (Yang 2007). We compared the one ratio model (1-ω) that assumes a constant dN/dS ratio (= *ω*, per site ratio of nonsynonymous -dN- to synonymous -dS- substitution) along tree branches (ωo), against a two-ratio model (2-ω) that assumes a different ratio for a given subclade (foreground = ωf) relative to the remaining sequences (background = ωb). In the genes *COL/COL4,* the test was implemented in the clades *COL*, *MonGHD7L* and *MonCOL4*, using the B-box I, B-box II, and the CCT domain together*.* In the *FD* gene lineage, comparisons were made for *MonFDL1, OrchFDL2A, OrchFDL2B,* including all the bZIP and SAP domains*.* In the *FLC/FUL* gene lineage tests were performed for *EudiFLC, MonFLC, EudiAP1/FUL, VRN1, MonFUL1* and *MonFUL2,* using only the conserved MIK domains of these *MADS-box* genes. Finally, in the *SOC1* gene lineage tests were implemented in the *EudiAGL42/71/72, EudiAGL14/19, EudiSOC1/AGL20, OrchSOC1L 1A, OrchSOC1L 1B* and *OrchSOC1L 2* clades, including all four MIKC domains.

### Identification of conserved motifs across angiosperms

In order to identify protein domains previously reported and new conserved motifs for each *COL/COL4, FD, FLC/FUL* and *SOC1* proteins, permanently translated CDS were used as input on the MEME server (meme-suite.org/tools/meme; (Bailey et al. 2009, 2015)). The sampling included 159 sequences of COL/COL4 proteins, 87 from FD proteins, 113 from FLC/FUL proteins and 108 from SOC1 proteins. Motif search was done using default settings and different numbers of conserved motifs were selected and numbered according to the protein family.

### Morpho-anatomical characterization of the flowering transition in *Elleanthus aurantiacus*

In order to establish changes in size, and the initiation of lateral organs as well as new morphological features occurring during flowering transition in the selected orchid species *E. aurantiacus* light microscopy (LM) and scanning electron microscopy (SEM) were used. For SEM analysis, SAM, IM and FBs from *E. aurantiacus* were collected in 70% ethanol and stored for one month or longer. Apices and buds were dissected in ethanol 90% following (Madrigal et al. 2021; Ospina-Zapata et al. 2020). The dissected samples were dehydrated in a progressive ethanol series. Samples and were critical point-dried using a Baltec CPD 030, coated with pure gold using an Emitech K550 sputter coater. Finally, samples were examined and photographed at 10 kV on a Zeiss SUPRA 40VP scanning electron microscope. For anatomical analyses, SAM and IM buds in different developmental stages were prepared by conventional dehydration with ethanol and histochoice (VWR, Radnor, USA) using standard series. Samples were embedded in Paraplast Plus (Leica Biosystems, Buffalo Grove, USA) and were sectioned at 10 µm with a Leica RM2125 RTS microtome. Sections were stained in safranin and astra-blue and examined using a Zeiss Primo Star Compound Microscope equipped with an Axiocam ERc 5 s Zeiss digital camera with Zen 2.3 Lite software.

### RT-PCR expression analysis of GRN candidate genes

In order to assess the expression patterns of the *COL/COL4, FD, FLC/FUL* and *SOC1* homologs in orchids, *Elleanthus aurantiacus* was selected. The reasons for such selection are the following: (1) it is a neotropical terrestrial orchid, easily accessible in the field, (2) it flowers two times per year in tune with the rainy seasons, (3) it had a low copy number of all tested gene lineages, and (4) the availability of plant material in vegetative and reproductive stages was sufficient to record morphological changes during reproductive transition (see below). Dissections of vegetative (SAM) and inflorescence (IM) meristems, flower buds (FB) and leaves (L) were made following the landmarks observed in the morpho-anatomical analysis, and were collected in liquid nitrogen separately. Total RNA extraction from each dissected tissue was done using TRIsure (Bioline, London, UK) according to manufacturer instructions and it was resuspended in 20 μl of miliQ water. The RNA was treated with DNAseI (Invitrogen, Waltham, USA) and was quantified using NanoDrop 2000 (Thermo Scientific, Waltham, USA). A total of 3.0 μg of RNA was used for cDNA synthesis using SuperScript III Reverse Transcriptase (Invitrogen, Waltham, USA). Specific primers were designed for the amplification of each isolated gene of interest (GOI) avoiding conserved domains (Supplementary Table S2). The amplification reactions of each GOI were done using 6.0 μl of EconoTaq (Lucigen, Middleton, USA), 4 μl of nuclease-free water, 1 μl of bovine serum albumin (5 μg/ml), 1 μl of Betaine (5 μg/ml), 1 μl of forward primer, 1 μl of reverse primer, and 1 μl of cDNA, for a total of 15 μl. The genes were amplified by PCR during 30 cycles following the next thermal profiles: an initial denaturation step (94 °C for 30 s), an annealing step (two degrees under the annealing temperature -Tm of primer with less Tm, for 30 s) and one extension step (72 °C for 40 s). All reactions finished with a final elongation step (72 °C, during 10 min) and a cold incubation (4° forever). *ACTIN* was used as a positive control. The amplicons were visualized on 1% agarose gel with ethidium bromide and digitally photographed using a Whatman Biometra ® BioDoc Analyzer. For comparative purposes, a review of the reported expression patterns was carried out in *Arabidopsis thaliana, Oryza sativa* and* Zea Mays* available in the BAR database (http://bar.utoronto.ca/), *Apostasia schenzhenica, Dendrobium catenatum* and *Phalaenopsis equestris* available in the OrchidBase 4.0 database (http://orchidbase.itps.ncku.edu.tw/est/home2012.aspx) and *Phalaenopsis aphrodite* available in the Orchidstra 2.0 database (http://orchidstra2.abrc.sinica.edu.tw/orchidstra2/index.php).

### RNA-seq experiments in dissected SAM and IM of *Elleanthus arantiacus*

To compare the transcriptional differences between meristems during flowering transition de novo transcriptomes from *E. aurantiacus* were obtained from the dissected vegetative meristem (SAM) and reproductive meristem (IM). Freshly dissected tissue was pooled from different plants at the same developmental age and stage in order to avoid biases caused by a plant. The experiment was conducted with three biological replicates per stage. All tissue was ground using liquid nitrogen. Total RNA was extracted using TRIsure (Bioline, London, UK) according to manufacturer instructions. The RNA-seq experiments were conducted using Truseq mRNA library construction kit (Illumina, USA) and sequenced on a NovaSeq 6000 equipment (Illumina, USA) with paired end readings of 100 bp. Different libraries were done independently for each RNA extraction. Read cleaning was performed with PRINTSEQ-LITE with a quality threshold of Q30 and contig assembly was computed using Trinity package following default settings (Pabón-Mora et al. 2023). The transcriptome assembly was performed for each SAM and IM dissections, and a combined transcriptome was also included (Supplementary Table S3). Orthologous gene search was performed using BLASTN with the orchid homologs *COL/COL4, FD, FLC/FUL* and *SOC1* as query. To estimate the relative abundance of the assembled contigs, cleaned reads were mapped against the de novo assembled dataset using the algorithm Kallisto with default settings (https://pachterlab.github.io/kallisto/). Kallisto quantifies transcript expression normalizing the relative abundance of each contig/transcript using the transcript per million (TPM) metrics (Owens et al. 2019; Pabón-Mora et al. 2023). The heatmap was constructed using the R package pheatmap (https://cran.r-project.org/web/packages/pheatmap/index.html).

## Results

### *CONSTANS-Like/CONSTANS-Like 4 (COL/COL4) *gene evolution

An exhaustive search in angiosperms for members of the B-box zinc finger protein family (BBX) group I including *CONSTANS Like/CONSTANS Like 4 (COL/COL4)* genes was made. The search resulted in the isolation of 359 homologs belonging to 120 species from flowering plants (Supplementary Table S1), of which, 30 homologs belong to 9 species of early diverging angiosperms, 15 are from 7 species of basal eudicots, 65 are from 22 species of core eudicots, 90 are from 38 species of non-orchid monocots and finally, 159 correspond to 44 species of Orchidaceae. Nucleotide sequences of all isolated homologs were used in the maximum likelihood (ML) phylogenetic analysis and the homolog of *COL9* from *Amborella trichopoda* (*AmtrgCOL9*) was used as outgroup.

**Fig. 1:**
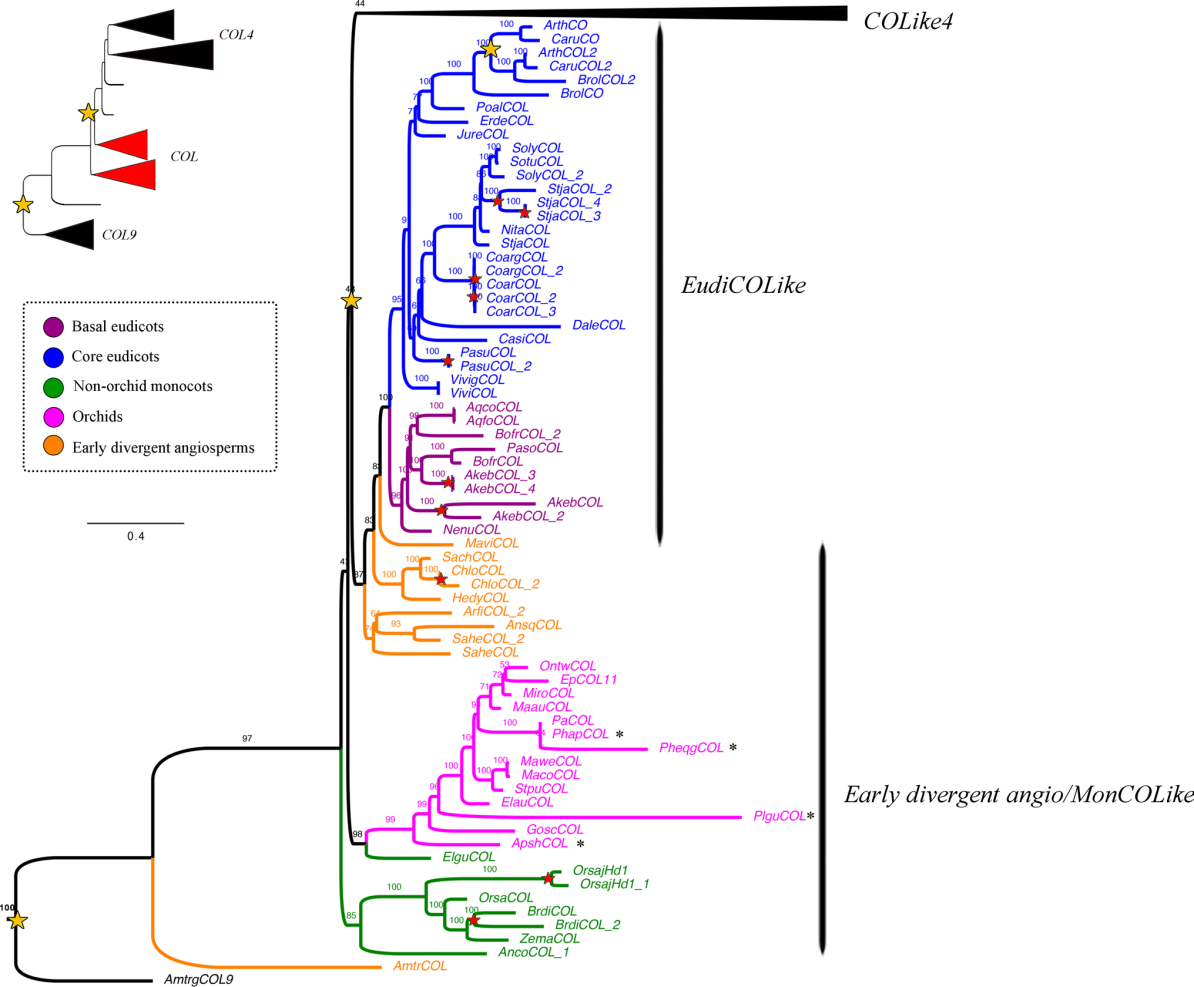
ML analyses of the *CONSTANS Like/CONSTANS Like 4* gene lineages in angiosperms, expanded in the *CONSTANS Like* homologs. Summary tree (upper left), the expanded clade in the figure is indicated in red. Tree branch and taxa colors follow the conventions on the left. Yellow stars indicate large-scale duplication events, red stars represent species-specific duplications. The numbers in each node indicate the Ultrafast Bootstrap (UFBS) values. The asterisks indicate sequences isolated from orchid genomes available. The collapsed clade corresponds to *COL4* homologs (see Figs. 2 and 3). Tree branch colors follow the conventions in the dotted line on the upper left side. Scale: 0.4

**Fig. 2:**
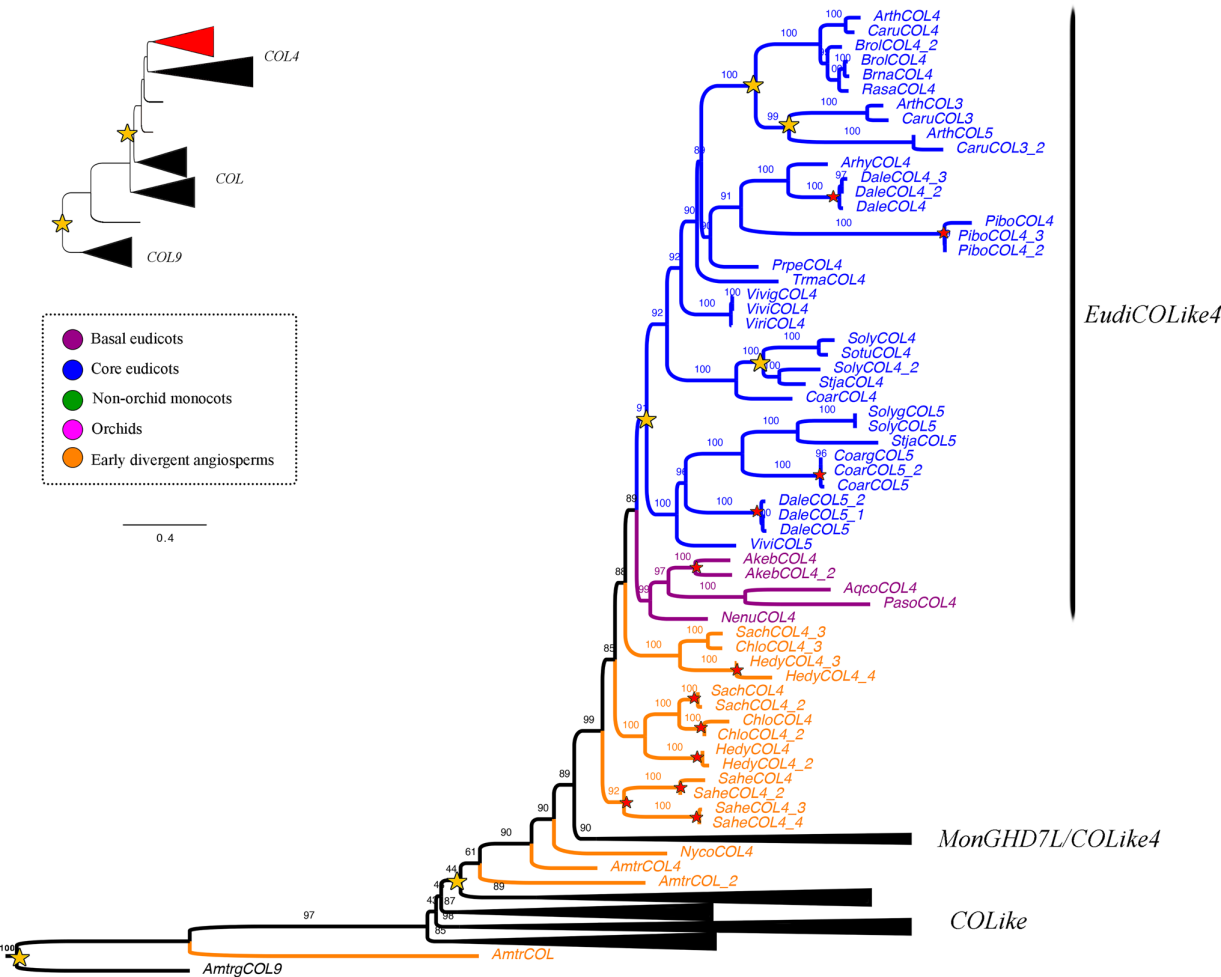
ML analyses of the *CONSTANS Like/CONSTANS Like 4* gene lineages in angiosperms expanded in the *EudiCOL4* genes. Summary tree (upper left), the expanded clade in the figure is indicated in red. Tree branch and taxa colors follow the conventions on the left. Yellow stars indicate large-scale duplication events, red stars represent species-specific duplications. The numbers in each node indicate the Ultrafast Bootstrap (UFBS) values. The asterisks indicate sequences isolated from orchid genomes available. The collapsed clades correspond to *COL* homologs (see Fig. 1) and *MonCOL4/GHD7L* homologs (see Fig. 3). Tree branch colors follow the conventions in the dotted line on the upper left side. Scale: 0.4

**Fig. 3:**
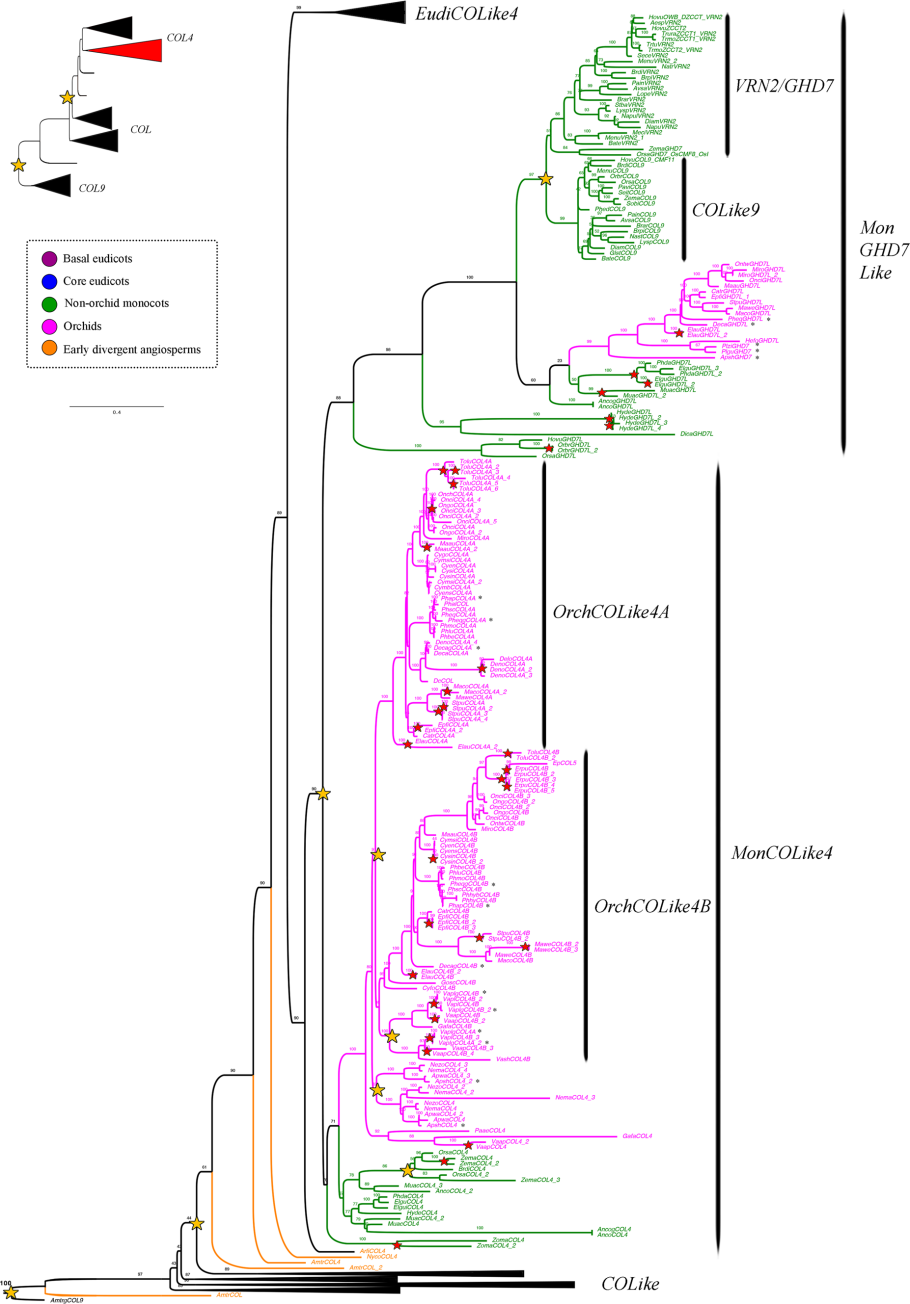
ML analyses of the *CONSTANS Like/CONSTANS Like 4* gene lineages in angiosperms expanded in the *MonCOL4/GHD7L* genes. Summary tree (upper left), the expanded clade in the figure is indicated in red. Tree branch and taxa colors follow the conventions on the left. Yellow stars indicate large-scale duplication events, red stars represent species-specific duplications. The numbers in each node indicate the Ultrafast Bootstrap (UFBS) values. The asterisks indicate sequences isolated from orchid genomes available. The collapsed clades correspond to *COL* homologs (see Fig. 1) and *EudiCOL4* homologs (see Fig. 2). Tree branch colors follow the conventions in the dotted line on the upper left side. Scale: 0.4

Our analysis showed two large-scale duplication events prior to angiosperm diversification that separate *COL9* (outgroup) (UFBS = 97), from paraphyletic *COL* genes (UFBS = 43, UFBS = 48, UFBS = 44) and monophyletic *COL4* genes (UFBS = 90, Figs. 1, 2, 3). *COL* genes are primarily retained as single copy, except in the Brassicaceae, as a result of a local duplication in the family (UFBS = 100, Fig. 1). Intra-specific duplications can be detected in *Akebia quinata, Brachypodium distachyon, Chloranthus spicatus, Coffea arabica, Oryza sativa, Paeonia suffruticosa,* and* Streptosolen jamesonii.*

Conversely, the phylogenetic history of the *COL4* genes is marked by reiterative duplications. At least 4 duplication events have occurred in the eudicots, labelled here as *EudiCOL4* (Fig. 2), one prior to the diversification of core eudicots (UFBS = 91), two duplications specific to Brassicaceae (UFBS = 100 and UFBS = 99), and one in Solanaceae (UFBS = 100). Intraspecific duplications have occurred in *Akebia quinata, Chloranthus spicatus, Coffea arabica, Dalea cuatrecasasii, Hedyosmum goudotianum* and *Sarcandra chloranthoides*. Different to *EudiCOL4*, their homologs in monocots have undergone several duplication events (Fig. 3). An early duplication in monocot diversification resulted in the *MonGHD7Like* (UFBS = 88) and *MonCOLike4* (UFBS = 76) clades. Additional local duplications in Poales result in the previously reported *VRN2/GHD7* (UFBS = 51) and *COLike9* (UFBS = 99) paralog clades (Woods et al. 2016). Finally, three additional duplications have occurred in the Orchidaceae for *MonCOLike4* homologs, one specific to the Apostasioideae subfamily (UFBS = 92), one specific to the Vanilloideae subfamily (UFBS = 100), and another prior to Vanilloideae diversification, resulting in the *OrchCOLike4A* (UFBS = 100) and *OrchCOLike4B* (UFBS = 88) clades. Species-specific duplications in the *MonCOL4/GHD7L* clades are recorded in *Apostasia wallichii, Cymbidium sinense, Dendrobium nobile, Elaeis guineensis, Elleanthus aurantiacus, Epidendum fimbriatum, Erycina pusilla, Hypoxis decumbens, Masdevallia coccinea "*alba*", Masdevallia wendlandiana, Maxilaria aurea, Musa acuminata, Oncidium "*Gower Ramsey*", Oryza brachyantha, Stelis pusilla, Tolumnia “*Cherry red x Ralph yagh*”, Vanilla aphylla, Vanilla planifolia, Zea mays* and* Zoostera marina.*

We analyzed the protein sequences of COL/COL4 across flowering plants. These belong to the B-box zinc finger protein family (BBX) group I and contain two B-box domains (B-box I and B-box II) and a CCT domain (Gangappa and Botto 2014; Khanna et al. 2009). We found that the average length of COL proteins varies around of 226–396 aa whereas COL4 proteins range between 240–300 aa. Our analysis identified near the N-terminal region, the B-box I domain, corresponding to the conserved motif 3 and the more variable motifs 7 or 18 (Supplementary Figure S1). The B-box II domain corresponds in the analysis to the highly conserved motif 2, and the CCT motif at the C-terminal region, includes a putative nuclear localization sequence (NLS), and corresponds to motif 1. Motifs 5, 6, 8, and 25 are shared in all COL/COL4 proteins, except MonGHD7L. All COL/COL4 proteins, except those in the VRN2/GHD7 group, also share motif 9. However, it is possible that these motifs have remained undetected because of the inclusion of incomplete sequences in the analyses. Our analysis identified MonGHD7L exclusive motifs, namely motifs 14, 20, 22, as well as COL9 exclusive motifs, namely 12, 21, and 23. MonCOL4 proteins can be characterized by motifs 4, 7, 10, 11, 15, 16, 28 and 30. Similarly, motifs 24 and 29 are exclusive to OrchCOL4A and motif 27 is specific to Poaceae proteins inside MonCOL4. Finally, we identified COL specific motifs, namely 13, 16, 17 and 19, and the grass specific motif 26.

To determine if there are differences in selection constraints acting on the *MonCOL4/MonGHD7L* and *COL* proteins, a series of targeted likelihood ratio tests (LRT) were performed. First, a one ratio model was tested for all *MonCOL4/MonGHD7L* and *COL* sequences resulting in a ωo = 0.0519 (Supplementary Table S4). Next a two-ratio model was implemented to test shifts in selection rates in selected gene clades when compared to the remaining sequences. For this we used the B-box I, the B-box II, and the CCT domains. The test showed that all clades are under purifying selection, however, it was shown to be increased in *COL* (ωf = 0.0580 vs. ωb = 0.1792) and relaxed in the *MonCOL4* (ωf = 0.4761 vs. ωb = 0.1692)*,* and the *MonGHD7L* (ωf = 0.2832 vs. ωb = 0.1680) clades (Supplementary Table S4). Such relaxed purifying selection in *MonCOL4* coincides with large variation in sequence and the occurrence of unique protein motifs (i.e., motifs 4, 7, 10, 11, 15, 16, 28 and 30). Similarly, relaxed purifying selection in *MonGHD7L* coincides with shorter proteins and the loss of a number of conserved motifs (i.e., motifs 2, 3, 18) (Supplementary Figure S1).

### *FLOWERING LOCUS D (FD)* gene evolution

**Fig. 4:**
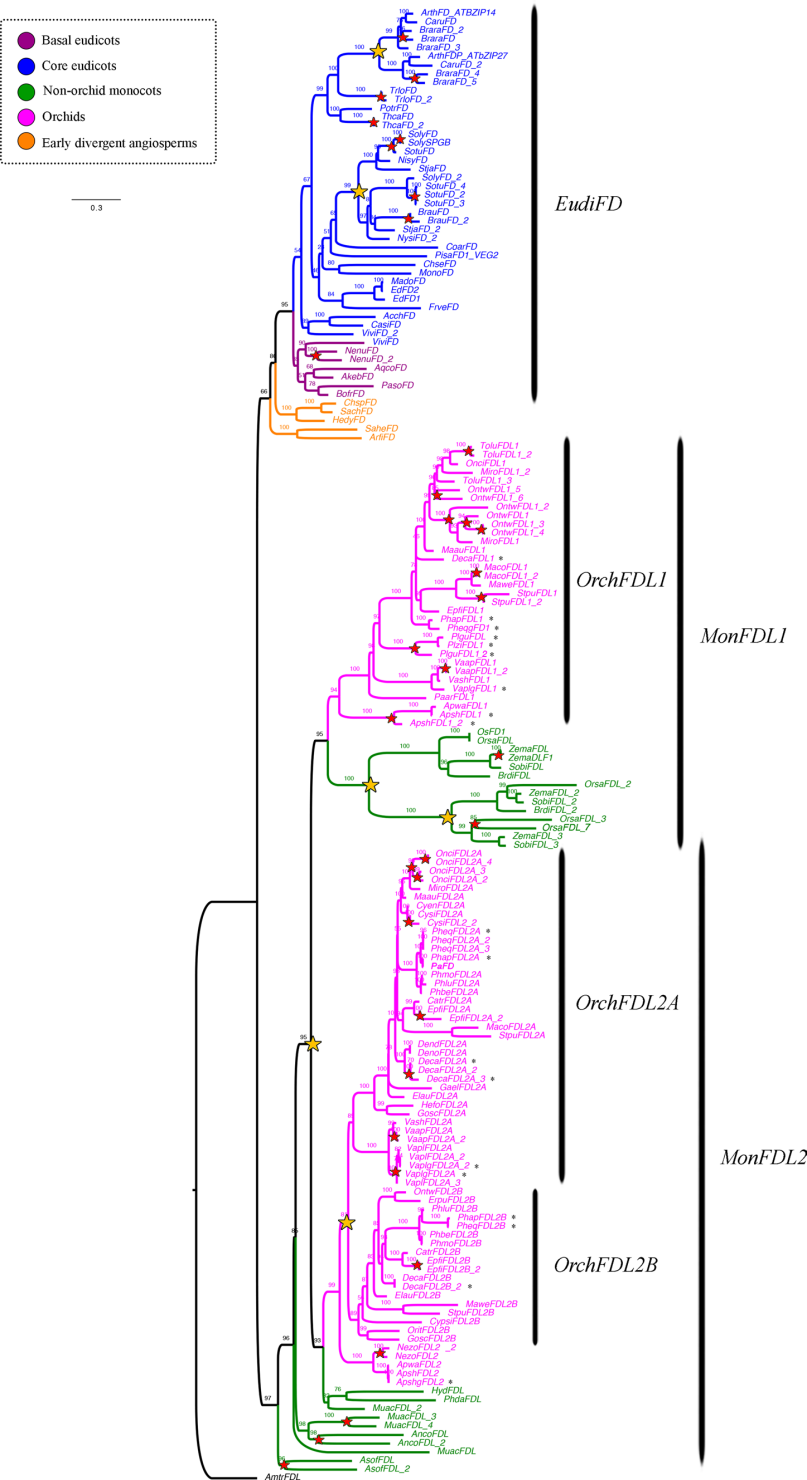
ML analyses of the *FLOWERING LOCUS D* genes. Tree branch and taxa colors follow the conventions on the left. Yellow stars indicate large-scale duplication events, red stars represent species-specific duplications. The numbers in each node indicate the Ultrafast Bootstrap (UFBS) values. The asterisks indicate sequences isolated from orchid genomes available. Tree branch colors follow the conventions in the dotted line on the upper left side. Scale: 0.3

Our sampling of the *bZip FD* genes includes 170 homologs from 78 angiosperm species, from which, 6 sequences are from 6 species of early divergent angiosperms, 4 from 4 species of basal eudicots, 41 from 22 species of core eudicots, 25 from 11 species of non-orchid monocots and finally, 94 correspond to 35 species belonging to Orchidaceae (Supplementary Table S1). We used the *Amborella Trichopoda, AmtrFDL,* as outgroup. The topology of the ML analysis (Fig. 4) shows a duplication prior to the diversification of Brassicaceae (UFBS = 100) and another one specific to Solanaceae (UFBS = 99) within *EudiFD*. Species-specific duplications are found in *Brassica rapa, Brunfelsia australis, Nelumbo nucifera, Solanum tuberosum* and *Theobroma cacao*. On the other hand, our topology recovered a duplication event prior to monocots forming the *MonFDL1* (UFBS = 95) and *MonFDL2* (UFBS = 93) genes. The former is recovered as a clade, whereas the latter is only rescued as a grade. Another duplication event within *MonFDL2* in the Orchidaceae, results in the *OrchFDL2A* (UFBS = 89) and *OrchFDL2B* (UFBS = 89, Fig. 4) paralogs. Two additional duplications have occurred in the Poaceae *MonFDL1* genes (UFBS = 100 and UFBS = 100). In other words, the duplication event prior to monocot diversification was followed by the retention of only one copy of *MonFDL1* in Poaceae, where all genome sequences of Poales species are nested, and *MonFDL2* in other non-orchid monocots, where we can find genome sequences from *Musa acuminata*, *Ananas comosus* and *Asparagus officinalis* (Fig. 4). Finally, intraspecific duplications have occurred in *Ananas comosus, Apostasia shenzhenica, Asparagus officinalis, Cymbidium sinense, Dendrobium catenatum, Epidendrum fimbriatum, M. acuminata, Masdevallia coccinea, Neuwiedia zollingeri, Oncidium "*Gower Ramsey*", Oncidium "*Twinkle*", Phalaenopsis equestris, Platanthera guangdongensis, Stelis pusilla, Tolumnia “*Cherry red x Ralph yagh*”, Vanilla aphylla,* and *Vanilla planifolia*.

FD belongs to the bZIP family characterized by the bZIP at the C-terminal and a SAP motif targeted by calcium-dependent protein kinases (CDPKs) important for FD functions (Abe et al. 2005). The FD protein sequence analysis across flowering plants showed that all protein sequences have an average length of 130-300aa. In our analysis the bZIP domain and the SAP motif correspond to motifs 1 and 6, respectively. Additional conserved motifs across angiosperms include motifs 2, 3, 4, 5, 7 and 17 (Supplementary Figure S2). EudiFD proteins share motif 18. Conversely, monocot proteins have a larger variation in motif number. Some MonFDL1 and OrchFDL2A proteins replace conserved motif 2 by a more variable motif 13. Poaceae proteins often lack motifs like 3, 4, 7 and 17, and instead have the specific motifs 15 and 20. Similarly, MonFDL1 proteins lack the motifs 5, 7 and 17 and have specific motif 11. Finally, MonFDL2 proteins share motifs 8, 10, and 12, and within diagnostic motifs 9 and 14 can be used to recognize OrchFDL2A, while motifs 16 and 19 can be used to recognize OrchFDL2B proteins (Supplementary Figure S2).

Likelihood ratio tests (LRT) determined important variation in selection constraints acting on FD genes (Supplementary Table S4). A one ratio model was tested for all *FD* sequences resulting in a ωo = 0.0332. Next a two-ratio model was implemented to test shifts in selection rates in *MonFDL1*, *OrchFDL2A* and *OrchFDL2B* when compared to the remaining sequences. For this we used the bZIP and SAP domains. The test showed that all clades are under strong purifying selection (ω_0_ = 0.0332) (Supplementary Table S4). However, the analyses showed that the purifying selection is relaxed in *OrchFDL2A* (ωf = 0.1553 vs. ωb = 0.0527) and O*rchFDL2B* (ωf = 0.0799 vs. ωb = 0.0421) which coincides with extensive sequence variation (i.e., in motifs 8, 10, 12). On the other hand, an increased purifying selection was found in *MonFDL1* (ωf = 0.0053 vs. ωb = 0.0502) (Supplementary Figure S2).

### *FLOWERING LOCUS C/FRUITFULL (FLC/FUL) *gene evolution

**Fig. 5:**
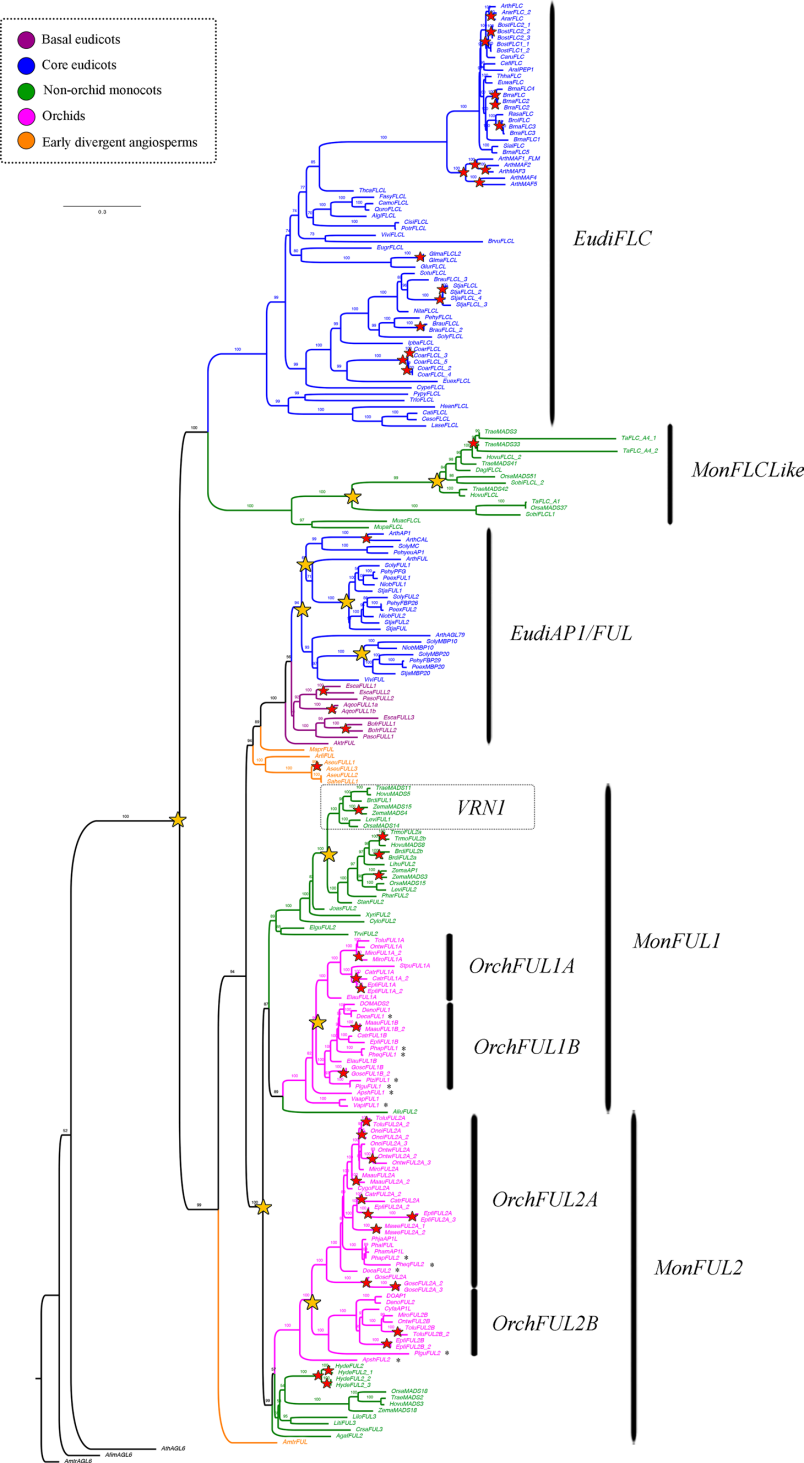
ML analyses of the *FLOWERING LOCUS C/FRUITFULL* genes. Tree branch and taxa colors follow the conventions on the left. Yellow stars indicate large-scale duplication events, red stars represent species-specific duplications. The numbers in each node indicate the Ultrafast Bootstrap (UFBS) values. The asterisks indicate sequences isolated from orchid genomes available. Tree branch colors follow the conventions in the dotted line on the upper left side. Scale: 0.3

With an exhaustive sampling we are able to isolate 230 *MADS-box* type II *FLC/FUL* homologs from 108 Angiosperm species, including 9 sequences of 5 species of early divergent angiosperms, 10 from 5 basal eudicots, 92 from 45 core eudicots, 53 from 26 from non-orchid monocots and finally, 66 correspond to 27 species from Orchidaceae (Supplementary Table S1). We used *AGL6* genes from* Amborella trichopoda (AmtrAGL6), Aristolochia fimbriata (AfimAGL6)* and *Arabidopsis thaliana (AthAGL6)* as outgroup*.* The analyses recovers the sister group relation already known between *FLC* and *AP1/FUL* genes. The resulting phylogenetic tree confirms that *FLC* genes are lacking in orchids, while they are still present in Poaceae, and are extensively diversified in eudicots (Fig. 5). We confirm this with an exhaustive search in all the orchid genomes available without a successful isolation of homologs. Two specific duplication events have occurred within *FLC* genes in grasses (UFBS = 100 and UFBS = 99), and intraspecific duplications are found in *Arabidopsis arenosa, Arabidopsis thaliana, Boechera stricta, Brassica rapa, Brunfelsia australis, Coffea arabica, Glycine max, Streptosolen jamesonii* and *Triticum aestivum*.

On the other hand, *FUL* genes have undergone at least two duplication events in core eudicots resulted in the *AGL79* (also called *euFULII*, UFBS = 93), *FUL* (also called *euFULI*, UFBS = 71) and *AP1/CAL* (UFBS = 99) homologs (Fig. 5, (Maheepala et al. 2019)). Our analysis recovered the reported additional duplications in Solanaceae, one inside eu*FULI* (UFBS = 100) and the other one within *euFULII* genes (UFBS = 100). One additional duplication event has occurred within monocots, resulting in the *MonFUL1* (UFBS = 87) and *MonFUL2* (UFBS = 99) clades. *MonFUL1* includes the previously reported *VRN1* and *FUL2* genes in grasses (McKeown et al. 2016; Preston and Kellogg 2006, 2007). Interestingly, our topology supports that *VRN1* is the result of a specific duplication inside Pooideae (UFBS = 100) and orchids only have the pre-duplication copies. On the other hand, *MonFUL2* includes the reported *FUL3* genes in grasses and *FUL-like* in non-grass monocots (Preston and Kellogg 2006). Two additional duplication events have been found in *MonFUL1* and *MonFUL2,* inside Orchidaceae as previously reported (Valoroso et al. 2019), namely here as *OrchFUL1A*—*OrchFUL1B* and *OrchFUL2A*—*OrchFUL2B* (Fig. 5). We confirm the reliability of those duplications using the genomic information available for orchids. Additionally, species-specific duplications were found in *Aquilegia coerulea, Arabidopsis thaliana, Asarum europaeum, Bocconia frutescens, Brachypodium distachyon, Cattleya trianae, Epidendrum fimbriatum, Eschscholzia californica, Gomphichis scaposa, Hypoxis decumbens, Masdevallia wendlandiana, Maxilaria aurea, Miltoniopsis roezlii, Oncidium ´*Twinkle*´, Tolumnia ´*Cherry red x Ralph yagi*´, Triticum monococcum* and *Zea mays.*

FLC/FUL proteins belong to the MADS-box type II gene family and all the sequences show the presence of the typical MIKC domains present in MADS-box proteins (Supplementary Figure S3, Par̆enicová et al. 2003; Smaczniak et al. 2012a, 2012b)). Our protein sequence analysis found an average length of 193–210 aa for FLC proteins in eudicots and a slight reduction in size to 153–170 aa in monocot proteins. FUL proteins range in size between 240–260 aa across angiosperms. The MADS domain, conserved in all sequences, is here recovered in motifs 1 and 5. Domain I corresponds to motifs 3 in FUL sequences, 12 in MonFLC sequences and 14 in EudiFLC sequences. Domain K corresponds to motif 4 with 8 inside FLC and motif 2 in FUL proteins. FLC proteins have fewer diagnostic motifs in the C-terminal region, namely motif 20 in EudiFLC. Moreover, all MonFLC proteins have lost motif 4 in the C-terminal region (Supplementary Figure S3). Conversely, FUL proteins have more variation in C-terminal region. All FUL sequences have motifs 6, 7 and 10. Motif 6 corresponds to the canonical motif LLPAWML (Pabón-Mora et al. 2013; Pabon-Mora et al. 2014). The eudiAP1/FUL clade shares motif 15. The MonFUL proteins share motif 9. Motif 11 is specific to orchid MonFUL1, and Poaceae MonFUL1 shared motif 19. Furthermore, MonFUL2 have the diagnostic motifs 17 and 18.

To determine whether there were differences in selection acting on the different *FLC/FUL* clades, likelihood ratio tests (LRT) were carried out for the MADS, I and K domains. We were able to establish that both FLC and FUL genes are under purifying selection (ω_0_ = 0.4510) (Supplementary Table S4). However, strengthening degrees of purifying selection can be detected in *EudiFLC* (ωf = 0.4086 vs. ωb = 0.5102) and *EudiAP1/FUL* (ωf = 0.3761 vs. ωb = 0.4752)*.* Nevertheless, the degree of purifying selection is significantly relaxed in *MonFLC* (ωf = 0.6191 vs. ωb = 0.4344), *VRN1* (ωf = 0.4755 vs. ωb = 0.3742), *MonFUL1* (ωf = 0.9332 vs. ωb = 0.4390) and *MonFUL2* (ωf = 0.6250 vs. ωb = 0.3971), which coincides with variation in sequence and loss of some protein regions (i.e., loss of motifs 4, 6 and 8 in *MonFLC* genes). It also suggests a long-term functional maintenance in *EudiFLC* and *EudiAP1/FUL* and more diverging functions for the monocot counterparts, namely, in *MonFLC*, *VRN1, MonFUL1* and *MonFUL2* (Aagaard et al. 2006; Yang 2007).

### *SUPRESSOR of CONSTANS 1* (*SOC*) gene evolution

**Fig. 6:**
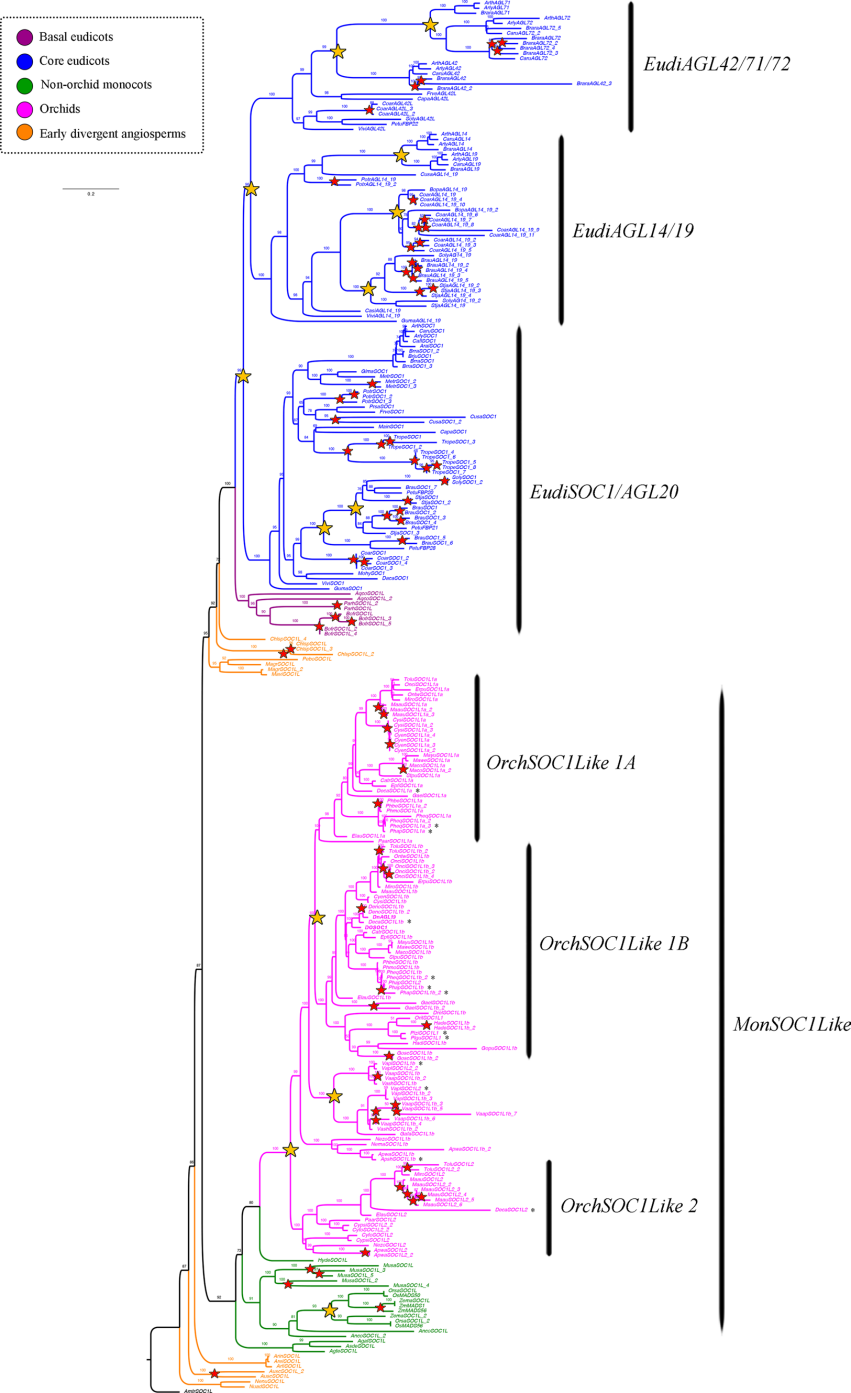
ML analyses of the *SUPPRESOR OF OVEREXPRESSION OF CONSTANS 1* genes. Tree branch and taxa colors follow the conventions on the left. Yellow stars indicate large-scale duplication events, red stars represent species-specific duplications. The numbers in each node indicate the Ultrafast Bootstrap (UFBS) values. The asterisks indicate sequences isolated from orchid genomes available. Tree branch colors follow the conventions in the dotted line on the upper left side. Scale: 0.2

Searches for members of the *MADS-box* type II *SOC1* genes resulted in 276 homologs from 94 species of flowering plants. These include 16 sequences from 11 species of early divergent angiosperms, 9 from 3 species of basal eudicots, 117 from 28 species of core eudicots, 19 from 9 species of non-orchid monocots, and finally, 115 correspond to 43 species belonging to Orchidaceae (Supplementary Table S1). We used *AmtrSOC1L* from *Amborella trichopoda* as an outgroup. Our ML topology found two duplication events prior to the core eudicot diversification giving rise to *EudiAGL42/71/72* (UFBS = 100), *EudiAGL14/19* (UFBS = 100) and *EudiSOC1/AGL20* (UFBS = 100, Fig. 6). Inside *EudiAGL42/71/72* two additional duplications have occurred in Brassicaceae (UFBS = 99 and UFBS = 100). Furthermore, within *EudiAGL14/19* local duplication events can be traced to the Rubiaceae (UFBS = 100), the Solanaceae (UFBS = 100) and the Brassicaceae (UFBS = 100). Finally, two additional duplications have occurred in *EudiSOC1/AGL20* inside Solanaceae (UFBS = 100 and UFBS = 100). Moreover, intraspecific duplications have found in *Bocconia frutescens, Brunfelsia australis, Brassica rapa, Chloranthus spicatus, Coffea arabica, Cucumis sativus, Medicago trunculata, Papaver rhoeas, Populus tremuloides, Solanum lycopersicum, Streptosolen jamesonii* and* Tropaeolum longifolium.*

In parallel, the phylogenetic tree recovers independent duplications in the monocot *SOC1* homologs (*MonSOC1L*). There is at least one duplication occurring prior to the Orchidaceae diversification resulting in the *OrchSOC1L1* (UFBS = 98) and *OrchSOC1L2* (UFBS = 100), and an additional duplication after Vanilloideae diversification forming the clades *OrchSOC1L1A* (UFBS = 99) and *OrchSOC1L1B* (UFBS = 100) (Fig. 6). A separate duplication event has occurred in Poaceae (UFBS = 93). Finally, intraspecific duplications are found in A*postasia wallichii, Austrobaileya scandens, Cymbidium ensifolium, Cymbidium sinense, Dendrobium nobile, Gastrodia elata, Gomphichis scaposa, Habenaria delavayi, Masdevalia coccinea, Maxilaria aurea, Musa acuminata, Oncidium "*Gower Ramsey*", Phalaenopsis aphrodite, Phalaenopsis bellina, Tolumnia ´*Cherry red x Ralph yagi*´, Vanilla aphylla, Vanilla planifolia* and* Zea mays.*

SOC1 proteins are MADS-box type II family members, hence the sequences show the presence of the typical MIKC domains and have an average length of 226–300 aa (Supplementary Figure S4, Par̆enicová et al. 2003; Smaczniak et al. 2012a, 2012b). The MADS domain is included in motifs 1 and 7 present in all SOC1 proteins. The I domain is recovered in motifs 3, 6 and with slight variations in motifs 10 or 14 in proteins of the MonSOC1L clade. The K domain is formed by motifs 2 and 5. At the C-terminal region, motif 4 is highly conserved and correspond to a diagnostic SOC1 sequence (DVETELYIGLP) within MADS-box proteins (Ding et al. 2013). Motifs 9 and 11 are specific to OrchSOC1L, and motif 13 is exclusive of OrchSOC1L1B. (Supplementary Figure S4).

To determine the selection acting on the different *SOC1* clades, likelihood ratio tests were carried out for all MIKC domains. We were able to establish that SOC1 genes are under strong purifying selection (Supplementary Table S4). In effect, all comparisons show a strengthening degrees of purifying selection: *EudiAGL42/71/72* (ωf = 0.0159 vs. ωb = 0.0526), *EudiAGL14/19* (ωf = 0.0121 vs. ωb = 0.0564), *EudiSOC1/AGL20* (ωf = 0.2208 vs. ωb = 0.0445), *OrchSOC1L 1A* (ωf = 0.0469 vs. ωb = 0.0483), *OrchSOC1L 1B* (ωf = 0.2208 vs. ωb = 0.0445) and *OrchSOC1L 2* (ωf = 0.1903 vs. ωb = 0.0466). All the above analyses suggest conserved roles in function in all SOC1 proteins (Aagaard et al. 2006; Yang 2007).

#### Expression analysis of flowering candidate genes

**Fig. 7:**
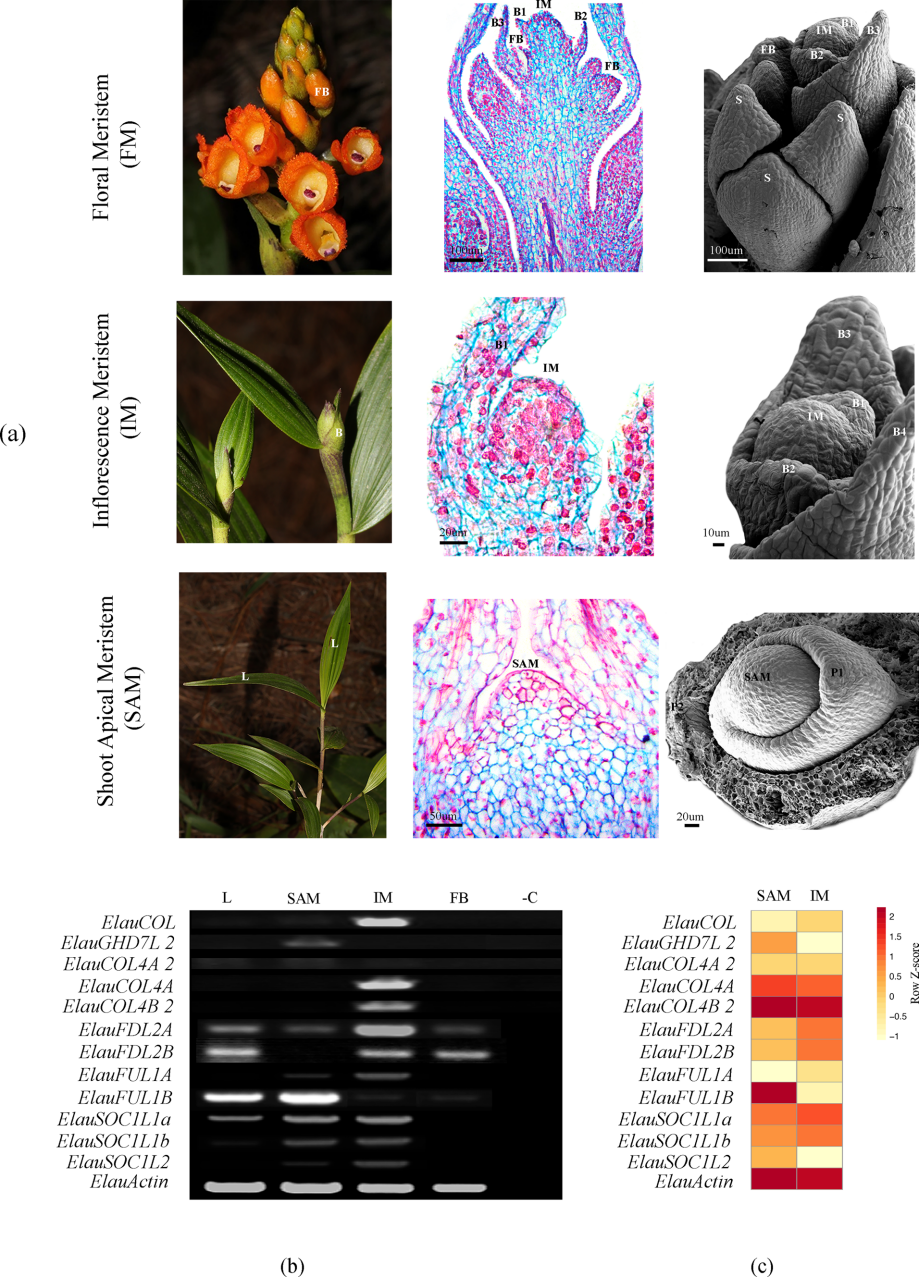
**a** Macroscopic field photographs and morpho-anatomical dissections to precisely identify correct stages to perform RT-PCR and RNAseq experiments during the phase transition in *Elleanthus aurantiacus*. From the bottom up are shown the vegetative meristem (SAM), the transitional inflorescence meristem (IM), and the floral meristem (FM). B: Bract; FB: Floral bud; IM: Inflorescence meristem; L: leaves; P: Plastochron; S: Sepal; SAM: Vegetative meristem. Scale is showed on top of the scale-bar. **b** RT-PCR expression patterns of *COL/COL4, FD, FLC/FUL* and *SOC1* genes in *E. aurantiacus* dissected organs, Actin was used as positive control. L: leaves, SAM: shoot apical vegetative meristem, IM: Inflorescence meristem and FB: Floral buds; -c: negative control lacking cDNA. **c** Heatmap expression data of *COL/COL4, FD, FLC/FUL* and *SOC1* genes in the SAM and IM dissected tissues. Genes are displayed in rows with normalized transcripts per million (TPMs) values for the comparisons. Gene expression levels follow the row z score convention at the top right, with dark red indicating up-regulation and bright yellow indicating down-regulation

We selected the terrestrial orchid *Elleanthus aurantiacus* for a complete flowering transition morpho-anatomical characterization followed by semi-quantitative RT-PCR and directed comparative transcriptomic analyses for all homologs of *CO/COL4, FD, FLC/FUL* and *SOC1.* The vegetative meristem (SAM) produces alternate leaves during almost two years. After floral induction, the axillary buds change their identity into a inflorescence meristem (IM) with a longitudinal enlarged dome capable of forming bracts in the flanks with axillary floral meristems. During the reproductive stage, the inflorescence meristems (IM) produces between 25 and 30 flowers per raceme (Fig. 7a). Because our goal was to record the expression patterns in different developmental stages during the vegetative to reproductive transition in this biannual flowering species, we dissected the SAM, IM, leaves and floral buds following Madrigal et al., (2021) (Fig. 7a). We isolated 7 *COL/COL4* genes, namely, *ElauCOL, ElauGHD7L, ElauGHD7L2, ElauCOL4A, ElauCOL4A2, ElauCOL4B* and *ElauCOL4B2*. However, a closer inspection shows that *ElauGHD7L* and *ElauCOL4B* could be the result of alternative splicing from their larger isoforms *ElauGHD7L2* and *ElauCOL4B2* respectively. In turn, only the expression of the latter two is showed. *ElauCOL* and *ElauCOL4A2* are expressed in leaves (L), the vegetative meristem (SAM) and the inflorescence meristem (IM). In contrast, *ElauGHDL2*, *ElauCOL4A* and *ElauCOL4B2* are restricted to the IM. The two isolated* FD* genes: *ElauFDL2A* and *ElauFDLB*, are expressed in the SAM and the IM, but *ElauFDL2A* can also be detected in leaves. *FUL* genes include *ElauFUL1A* and *ElauFUL1B* with high expression levels in L, SAM and IM, and low expression levels in floral buds (FB) of *ElauFUL1B*. Finally, there are three *SOC1* genes: *ElauSOC1L1a* and *ElauSOC1L1b* are expressed on L, SAM and IM, with low expression of *ElauSOC1L1b* in FB. Finally, *ElauSOC1L2* is expressed in both in SAM and IM (Fig. 7b). Directed searches in our parallel RNA-seq experiments derived from tree replicates of SAM and IM in *E. aurantiacus,* validate these same expression patterns. Here, we used the highest standards of transcript normalization, and we rescued the same trends for all genes evaluated, with the exception of *ElauCOL4A* and *ElauCOL4B2* that are highly expressed in both SAM and IM (Fig. 7c).

## Discussion

Most studies have focused on identifying the FGRN in model species like *Arabidopsis thaliana* or *Oryza sativa*, or in crops, like canola, corn, pea, and tomato, to mention a few; however, comparative studies across flowering plants are still lacking. Notably, such studies aimed to identify and characterize members of the FGRN are still incipient in one of the richest groups of incredibly diverse floral forms with ornamental potential: the orchids. Comprehensive phylogenetic studies aimed to characterize the genetic complements of the FGRN in the Orchidaceae have been done for the *FT* and *AGL24/SVP* gene lineages. In both cases, the occurrence of several independent duplication events in monocots and eudicots has hindered a straightforward (or a one to one) comparison of the well-characterized FGRNs to orchids (Madrigal et al. 2021; Ospina-Zapata et al. 2020; Ramirez-Ramirez et al. 2021). Nevertheless, these analyses have been instrumental to tackle the complexity of the FGRN in terms of copy number, expression patterns, and the ecological links to flowering inductive conditions. In an effort to analyze all the remaining players of the FGRN we performed comprehensive phylogenetic analyses of all other members of the FGRN across flowering plants, with a focus on orchids. Here we identify large-scale duplication events, changes in the protein sequences after such duplications, variation in the evolutionary rates of resulting paralogous clades and targeted expression of isolated homologs in different orchids. Altogether the data presented here lays down a better framework to assess gene function of a restricted number of homologs identified more likely playing key roles during the flowering transition.

### Numerous duplications with adaptable functional evolutionary trends in the *COL/COL4 *gene lineage

The B-box zinc finger protein family (BBX) is classified into five groups, of which, type I includes the *Arabidopsis CONSTANS* and *CONSTANS Like 1*—*COL5* homologs. They are characterized by having three conserved domains: two B-box domains that function in protein–protein interaction and transcription regulation (Chou et al. 2013; Gangappa & Botto 2014; Griffiths et al. 2003), and one CCT (CO, CO-like, TOC1) domain that interacts with DNA (Griffiths et al. 2003; Song et al. 2012; Tiwari et al. 2010). In *Arabidopsis* is the CCT domain of CO the one that binds directly to the *FLOWERING LOCUS T* (*FT*) promoter to induce flowering in long-days (Gangappa & Botto 2014; Tiwari et al. 2010). In addition to the duplication event that resulted in the *COL* and the *COL4* genes, our ML topology recovers additional duplications inside *COL4.* This is in contrast to *COL* genes which are predominantly retained as single copy (Figs. 1, 2, 3, see exceptions in Simon et al. 2015). Our analyses also showed that while *COL* genes are under strengthening purifying selection, the *COL4* homologs undergo relaxed purifying selection, which coincides with large variation in sequences, specifically inside monocots (Supplementary Table S2 and Figure S1).

The primarily single copy *COL* genes can function as photoperiodic sensors in long-day (LD) plants such as *A. thaliana*, neutral-day (ND) plants like *Rosa chinensis* (Balcerowicz 2021; Denoyes et al. 2020; Lu et al. 2020), or in short-day (SD) plants, such as *Chenopodium rubrum* (Drabešová et al. 2014), *Hordeum vulgare* (Campoli et al. 2012; Griffiths et al. 2003; Turner et al. 2005), *Oryza sativa* (Hayama et al. 2003; Yano et al. 2000), and *Solanum tuberosum* (González-Schain et al. 2012). Also, *COL* genes are linked to other day length-dependent developmental processes such as tuberization in potato (González-Schain et al. 2012), bud dormancy and metabolism in poplar (Böhlenius et al. 2006; Hsu et al. 2012), and lateral root formation and shoot branching in *Arabidopsis* (Datta et al. 2005). However, our sampling only recovered *COL* genes in a few members of the Epidendroideae and Apostasioideae, which suggests that *COL* genes are not actively transcribed in meristematic tissues, and perhaps do not play significant roles in the flowering processes of all orchid subfamilies. *COL* homologs have been characterized from *Erycina pusilla* (Chou et al. 2013), *Phalaenopsis hybrida* (Zhang et al. 2011)*, P. aphrodite* (Ke et al. 2020), *Oncidium ‘*Gower Ramsey*’* (Chang et al. 2011), and *Dendrobium crumenatum* (Kaewphalug et al. 2017). Heterologous overexpression of *Phalaenopsis COL* genes result in an early flowering phenotype under SD (Ke et al. 2020; Zhang et al. 2011). Interestingly, in the tropical species *D. crumenatum*, the *DcCOL* mRNA is accumulated in the dark in LD, ND, and SD, suggesting that the regulation of *DcCOL* is controlled in a circadian rhythm-dependent manner independent of light response, and that photoperiod is nor the only, neither the most critical, factor for floral induction in *Dendrobium* (Kaewphalug et al. 2017)*.*

Conversely, *COL4/GDH7* genes act as flowering repressors in both eudicots and monocots (Datta et al. 2005; Hassidim et al. 2009; Shrestha et al. 2014; Steinbach 2019). The canonical *AtCOL4*, *AtCOL3* and *AtCOL9* (in the *EudiCOL4* clade) do so by controlling the expression of *FT* and *SOC1* (Datta et al. 2005; Hassidim et al. 2009; Shrestha et al. 2014; Steinbach 2019). Orchids have greatly diversified their *COL4* genes, especially in contrast to other monocots like grasses or other non-orchid Asparagales, which unlike orchids have large duplications in the paralogous *GHD7-like* genes (Fig. 3). In fact, *COL4* copies were identified in all orchid subfamilies, and have duplicated once in Apostasioideae, once in Vanilloideae and on a separate event, prior to the diversification of all remaining Orchidaceae subfamilies. These large-scale duplications were supported by clear sequence divergence into different clades coming from all available orchid genomes. We also noticed an increase of species-specific duplications, however, since many sequences come from transcriptomes they could be instead splicing variants. Thus, species-specific duplications will require confirmation in the future when more genomes become available. Nevertheless, the fact that similar copy number is found in different vanilla species, some with and some without a reference genome is suggestive of true copy number increase in each taxa. In grasses, both *GHD7* and *COL4* homologs (especially those within the *VRN2/GHD7* clade) are important for day length sensing, and their repressive activity over flowering promoters under non-inductive photoperiods (Kikuchi et al. 2012; Shrestha et al. 2014; Wei et al. 2010; Xue et al. 2008; Yan et al. 2011). In plants with vernalization requirements, like wheat and barley, *VRN2* genes also act as flowering repressors (Woods et al. 2016). Reduction of barley *BdVRN2* expression results in rapid flowering and elevated expression of *BdFT* and *BdVRN1* (Distelfeld et al. 2009; Woods et al. 2016; Yan et al. 2004). Currently, no functional data is available for any *COL4* orchid homolog, but it is tempting to speculate that an increase in *COL4* gene copy number could be linked to a more sophisticated flowering repression in orchids, compared to other monocots, using active copies like *GHD7*, *OrchCOL4A* and *OrhCOL4B* (Fig. 7).

Finally, a close inspection of the available gene expression datasets for *COL/COL4* homologs in different angiosperms species was used as a proxy to narrow down putative roles of the two gene groups. Our hypothesis was that if genes were largely restricted to leaves and the SAM, they were more likely to be repressing flowering, while if they were broadly expressed and remained active in the inflorescence meristems and floral buds were more likely to function as flowering promoters. However, most *COL/COL4* homologs from *Arabidopsis*, maize, and rice, as well as in several orchids show broad expression in leaves, and the SAM, but are sometimes expressed in IM, and fewer times in flowers and even in seeds (Fig. 7, Supplementary Figure S5 and Tables S5–S8). Even directed searches of targeted genes in our RNA-seq experiments showed expression of *COL4* genes in both vegetative and reproductive stages (Fig. 7c). Altogether, the data point to highly conserved *COL* genes in angiosperms, and a larger extent of duplication and sequence changes linked with possible functional diversification in the *COL4* homologs, especially in orchids because their increased number of copies. However, expression patterns alone in this case, are not sufficient to postulate a repressive or promoting role in flowering for either of these genes.

### Orchids have at least three *FD* copies more likely acting as flowering promoters.

*FD* is a basic leucine zipper (bZIP) transcription factor able to interact with diverse PEBP protein family members. Precisely, its specific interactions can result in flowering activating FD-FT or flowering repressive FD-TFL1 complexes (Ahn et al. 2006; Hanano and Goto 2011; Kaneko-suzuki et al. 2018; Wigge 2005). Across angiosperms FD complexes control flowering time and floral homeotic genes downstream, such as *SOC1* (Jang et al. 2017; Smith et al. 2011)*, SQUAMOSA PROMOTER BINDING PROTEIN-LIKE3-5 (SPL3-5)* (Jung et al. 2016)*,* and *LFY* (Jung et al. 2016; Zhu et al. 2020) and *AP1/FUL* (Collani et al. 2019; Jang et al. 2017). We found that monocot species have two *FD* clades: *MonFDL1* and *MonFDL2* (Fig. 4), that are able to form FD-PEBP complexes. The maize *DELAYED FLOWERING1* (*DLF1,* belonging to *MonFDL1*), the wheat *FDL2/FDL6* (belonging to *MonFDL1),* the rice *OsFD1* and *OsFD4* (belonging to *MonFDL1),* and the orchid *PaFD* (belonging to *MonFDL2-OrchFDL2A*), can interact with FT homologs to promote flowering (Cerise et al. 2021; Jang et al. 2015; Li and Dubcovsky 2008; Meng et al. 2011; Muszynski et al. 2006; Taoka et al. 2011). Same transcription factors can interact with 14-3-3 and TFL1 proteins to antagonize FT protein during inflorescence development (Kaneko-suzuki et al. 2018). Similarly, copies OsFD2 and OsFD7 in rice (belonging to *MonFDL1)* form complexes involved in leaf development and panicle development, through the interaction with RFT (FT homolog) and Hd3a, respectively (Brambilla et al. 2017; Kaur et al. 2021; Tsuji et al. 2013). Interaction specificity relies upon the C-terminal region of FD, which contains the SAP motif targeted by calcium-dependent protein kinases (CDPKs). Moreover, it is well-established that phosphorylation is essential for the recognition of FD by 14-3-3 proteins and the formation of the flowering activation complex (Kaur et al. 2021; Taoka et al. 2011). Conversely, the unphosphorylated FD can interact with TFL1 via 14-3-3 proteins to prevent flowering and maintain the SAM (Collani et al. 2019).

Expression data of *MonFDL1* homologs show high activity in vegetative and reproductive phases in rice*,* maize and* Apostasia schenzhenica,* with high levels of expression in storage organs in the latter (Supplementary Figure S6, Table S8, Tsuji et al. 2013). *MonFDL2* homologs from orchids exhibit a more diverse range of expression patterns in roots (where available), leaves, SAM and IM (Fig. 7, Supplementary Figure S6 and Table S5–S8, (Tsuji et al. 2013)). Expression and functional analyses point to a likely conserved role of all monocot *FD* genes as flowering promoters as they maintain the ability to form complexes with 14-3-3 proteins and *FT* due to an intact SAP. However, other putative roles of *FD* in leaf development (Jang et al. 2017; Teper-Bamnolker and Samach 2005; Tsuji et al. 2013), stomatal opening (Kinoshita et al. 2011), inflorescence architecture (Endo-Higashi and Izawa 2011; Hiraoka et al. 2013; Kaneko-suzuki et al. 2018; Kaur et al. 2021; Lifschitz et al. 2006; Smith et al. 2011; Zhu et al. 2020), phytohormone signaling (Romera-Branchat et al. 2020) or storage organ development (Navarro et al. 2011; Teo et al. 2017) cannot be excluded for *FD* orchid homologs.

### The canonical *FLC *flowering repressors are lacking in Orchidaceae

*MADS-box* genes in the *FLC* clade are critical negative flowering regulators in *Arabidopsis thaliana* that act in response to seasonal cues. *FLC* represses flowering, and only an extended exposure to low temperatures or vernalization, can repress the transcription of *FLC* and promote flowering (Alexandre and Hennig 2008; Gu et al. 2013; Helliwell et al. 2015; Lin et al. 2005; Madrid et al. 2021). Moreover, FLC target specificity is conferred by its interacting partners, the cofactors of the MADS-box proteins heterodimers, and chromatin remodeling complexes that define the spatiotemporal expression of downstream targets (Gu et al. 2013; Madrid et al. 2021). *FLC* genes are present in Poales (*MonFLC*) where they can also repress flowering. However, the monocot *FLC* genes are characterized by divergent, short protein sequences (Madrigal et al. 2021; Ruelens et al. 2013). Our data confirm that *MonFLC* genes have important losses in the C-terminal region motifs in comparison con *EudiFLC*, accompanied by characteristic signals of relaxed purifying selection, yet repressive roles in flowering are conserved in distantly related species (Fig. 5, Supplementary Figure S3, Supplementary Table S4). Strikingly, we did not recover *FLC* homologs from any available orchid genome, neither from any orchid transcriptome. This finding suggests that in temperate orchids, or tropical ones with cold treatments underlying flowering promotion, other flowering repressors different from *FLC* have to be in place (Alexandre and Hennig 2008; Madrigal et al. 2021; Ruelens et al. 2013). Putative factors able to take on flowering repressive roles in response to temperature changes include *COL4, AGL24*/*SVP*, *SMZ/TOE3* or, less likely *FUL* (Chen and Dubcovsky 2012; Deng et al. 2011; Lee et al. 2013; Mathieu et al. 2009; Ramirez-Ramirez et al. 2021; Xie et al. 2021).

### The contribution of other *MADS-box* genes involved in flowering transition to orchid reproduction.

Other *MADS-box *genes play key pleiotropic roles during flowering. Such is the case of *AP1/FUL* homologs often linked to positive regulation of the flowering transition (Jiang 2022; Jiang et al. 2022), floral meristem identity (Balanzà et al. 2019; Ferrándiz et al. 2000; Martínez-Fernández et al. 2020; Pabon-Mora et al. 2012), and cold response in vernalization-sensitive grasses (Chen and Dubcovsky 2012; Li et al. 2019; Preston and Kellogg 2007, 2008; Xie et al. 2021; Yan et al. 2003). *FUL-like* genes are increasingly diversified in monocots where they form two clades: *MonFUL1,* that includes the previously reported grass homologs *VRN1* and *FUL2;* and *MonFUL2* (named as *FUL3* in Preston and Kellogg (2006); Fig. 5). *VRN1* is the result of a specific duplication inside grasses, so the presence of pre-duplication copies of *MonFUL1* in orchids reinforces the idea that the cold response mechanisms are very different from those occurring in grass seasonal species like barley, wheat or oat. In grasses, *VRN1* is induced by vernalization and accelerates the transition to reproductive development at the shoot apex down regulating the activity of *VRN2* in leaves (Chen and Dubcovsky 2012; Distelfeld et al. 2009; Preston and Kellogg 2008; Trevaskis et al. 2006; Yan et al. 2004). *VRN1* can be epigenetically modified by histone modification complexes similar to those that repress *FLC* (Oliver et al. 2009; Trevaskis et al. 2003, 2007; Yan et al. 2003). However, it is also established that *VRN1* genes are not essential for flowering in grasses and that other *FUL* homologs may be partially redundant in function during transition to flowering and during spikelet development (Li et al. 2019; Petersen et al. 2004; Preston and Kellogg 2006, 2008). The only functional study in orchids shows that early flowering occurs in response to overexpression of *DoAP1* (from the *MonFUL2* clade) from *Dendrobium* Chao Praya Smile in *Arabidopsis* (Sawettalake et al. 2017). In general, expression data available is broad and supports pleiotropic mostly promoter roles for all *FUL-like* genes in flowering transition in grasses (Supplementary Figure S8, (Preston & Kellogg 2008)), and in orchids (Fig. 6, Supplementary Tables S5-S8, (Goh and Yu 2000; Tian et al. 2013)). While the evolution of cold responsiveness in Poales seems to be related with the transition of Pooideae lineages from the tropics into temperate zones (Chen & Dubcovsky 2012; Woods et al. 2016), little is known about the ecological triggers and associated molecular mechanisms by which cold responses could have evolved in orchids.

Finally, the *MADS*-*box* gene *SOC1* constitutes another important hub in the regulatory network underlying floral timing and flower development (Immink et al. 2012). *SOC1* is regulated by the antagonistic *CO* and *FLC*, the former being a floral activator and the later behaving as a floral repressor (Lee and Lee 2010). *CO* activates *SOC1* mainly through *FT* (Lee and Lee 2010). *SOC1* together with *AGL24* can regulate their mutual transcription to integrate flowering signals in the shoot apex from several genetic pathways, including the GA pathway and the flower initiation via *LFY* activation (Liu et al. 2008; Torti & Fornara 2012). *SOC1* is repressed during flowering transition by *FLC* direct binding (Lee and Lee 2010). Conversely, *SOC1* is downregulated in flowers by negative autoregulatory loops of *MADS*-box proteins like AP1, SEP3 an AG, in which SOC1 represses its own expression in combination with AG and API (Immink et al. 2012). Other *SOC1-like* related genes such as *AGL42, AGL71* and *AGL72 (EudiAGL42/71/72)*, are also involved in the promotion of flowering seemingly acting through a gibberellin-dependent pathway (Dorca-Fornell et al. 2011). *XAANTAL2* (*EudiAGL14* /*19*) is necessary and sufficient to induce flowering through the activation of *AP1*, and its regulation is important in floral meristem maintenance and determinacy (Garcı et al. 2015). Our results point to reiterative *SOC1* gene duplications in eudicots, in contrast with the single clade in monocots, with duplications restricted only to Orchidaceae (Fig. 6). Expression of *SOC-1* genes, like in the case of *FUL-like* genes, is broad and includes almost all stages from SAM to IM to FM (Dorca-Fornell et al. 2011; Garcı et al. 2015; Lee et al. 2004; Liu et al. 2008; Tadege et al. 2003) (Supplementary Table S5-S8, Fig. 7; S9). However, purifying selection is the rule in this gene lineage, suggesting functional conservation across angiosperms, which extrapolating from known functional data more likely corresponds to a positive regulation of flowering (Supplementary Figure S4, Table S4, (Ezoe et al. 2021)). In fact *OsMADS50* (belonging to *MonSOC1L*) is an important flowering activator that controls various floral regulators in rice like *OsMADS14 (FUL-like)*, *OsMADS15 (FUL-like)* and Hd3a (Lee et al. 2004). Moreover, the few studies assessing function by heterologous expression of orchid *SOC-1* homologs in *Arabidopsis* do show early flowering (Liu et al. 2016), linked to the upregulation of *AGL24* and *LFY* (Ding et al. 2013) supporting the idea of a general role of *SOC1-like* genes as flowering promoters. However, endogenous roles of *SOC1* genes remain to be evaluated in orchids.

## Conclusion and remarks

**Fig. 8:**
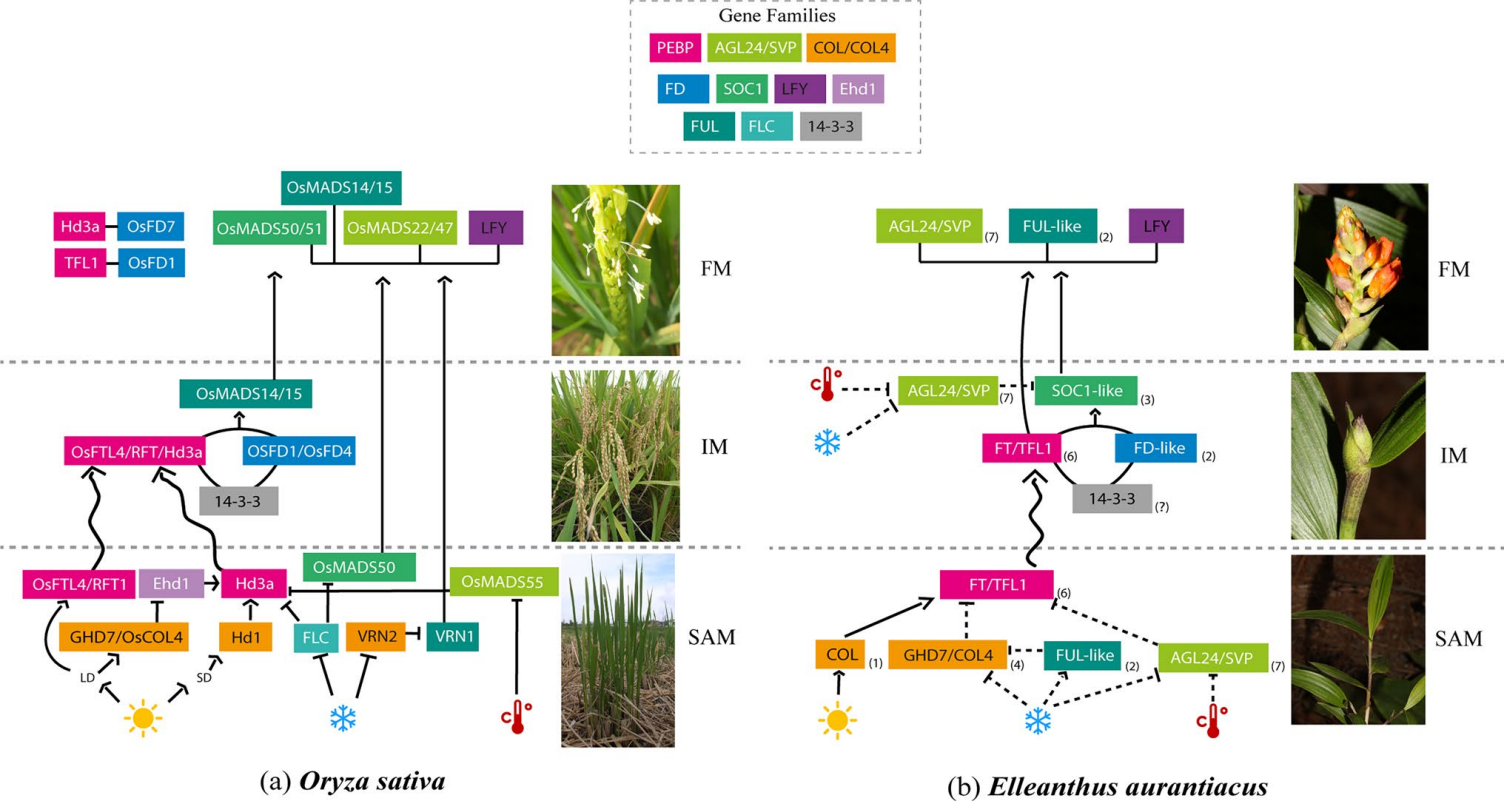
Flowering Genetic Regulatory Network (FGRN) in **a** *Oryza sativa* (Poaceae) in comparison with the proposed FGRN in **b** *Elleanthus aurantiacus* (Orchidaceae). Icons represent the flowering induction by photoperiod, vernalization (cold response) and ambient temperature. Color squares indicate different gene families. The rice FGRN was modified from (Chen & Dubcovsky 2012; Choi et al. 2014; Endo-Higashi & Izawa 2011; Fornara et al. 2008; Gu et al. 2022; Kaneko-suzuki et al. 2018; Kojima et al. 2002; Komiya et al. 2008; Lee et al. 2012; S. Lee et al. 2004; Preston & Kellogg 2008; Taoka et al. 2013; Taoka et al. 2011; Tsuji et al. 2013; Woods et al. 2016). *LFY/FLO*, *14–3-3* and *Ehd1* genes are not included in our analyses but are involved in flowering in rice (Doi et al. 2004; Nagasherre et al. 2008; Taoka et al. 2011). Dotted arrows indicate possible promoters or repressors in *E. aurantiacus*. The numbers in parentheses indicate the number of our isolated *COL/COL4, FD, FUL* and *SOC1* homologs, as well as, reported gene copy number from *AGL24/SVP* (Ramirez-Ramirez et al. 2021), *FT* (Ospina-Zapata et al. 2020) and *LFY* (Jang 2015). SAM: shoot apical vegetative meristem, IM: Inflorescence meristem, FM: Floral meristem. Rice plant photos by Matsuyuki (CC BY-SA 2.0) and Nandukambalapally (CC-BY-SA-4.0)

Gene duplication is the raw material for functional diversification and one of the key underlying mechanisms of emerging phenotypes and novel evolutionary features. In this study we analyzed the evolutionary history coupled with the available and new expression data of all flowering integrators Orchidaceae. Our data, together with previous data on orchid flowering regulators (Ospina-Zapata et al. 2020; Ramirez-Ramirez et al. 2021) and the information available from all orchid genomes, allowed us to conclude that: (1) The number of orchid homologs belonging to *PEBP, AGL24/SVP*, *COL4*, and *FUL* gene lineages is higher than in most other monocots including grasses due to orchid specific gene lineage duplications. The higher the copy number, the more likely it is for these gene lineages to diversify in function with respect to ancestral single copy genes. (2) Conversely, local duplications in Orchidaceae are mostly lacking in the *COL*, *FD* and *SOC1* gene lineages, which points to a retention of key functions under strong purifying selection in essential signaling pathways (Ezoe et al. 2021). (3) Expression patterns of most flowering integrators are broad and include both vegetative and reproductive stages, as a result expression data are important but insufficient to assign a putative for each gene copy during flowering. (4) Grasses and orchids are evolutionarily prone to gene duplication events, but these have occurred independently in the two lineages, thus it is difficult to readily extrapolate the functions identified to many flowering genes in rice, maize, wheat, and barley to orchids. Based on the available data we compare the FGRNs in the grass model *O. sativa* and the terrestrial orchid *Elleanthus aurantiacus.* This FGRN serves as a reference for future detailed spatio-temporal expression and functional analyses, that can incorporate the evaluation of all active copies and their association with environmental cues like ambient temperature and light (Fig. 8). The putative orchid FGRN presented here retains the most important genes involved in flowering transition in grasses, incluiding flowering promoters like *FT* homologs, *FD*, *COL*, *SOC1, FUL-like* and *LFY.* Our model also recovers *GHD7/COL4* and *AGL24/SVP* as the most likely flowering repressors in the absence of *FLC*. Finally, future validation of proposed model have to be done in order to asses to the interaction and represive or promotive function of the FGRN in different orchids with diverse habits, occupying distinct habitats.

### Author contribution statement

Yesenia Madrigal: Conceptualization, Formal analyses, Data curation, Writing–original draft, Review & editing, Funding. Juan F. Alzate: Resources, Data curation, Review & editing. Natalia Pabón-Mora: Conceptualization, SEM photographs, Resources, Supervision, Writing–review & editing, Funding. All authors approve the final manuscript.

### Supplementary Information

Below is the link to the electronic supplementary material.

Supplementary file1 (DOCX 74830 KB)

Supplementary file2 (XLSX 52 KB)
