# Supplementary material for: Evolution of major flowering pathway integrators in Orchidaceae
Source: Plant Reprod. 2023 Oct 12;37(2):85–109. doi: 10.1007/s00497-023-00482-7 (PMC11180029; doi:10.1007/s00497-023-00482-7)

**Supplementary Materials**

**Figure S1.**  Conserved motifs of COL/COL4 proteins across flowering plants identified with a MEME analysis. Each motif is represented by a coloured box with the corresponding summarized sequence at the right. Size of bar indicates size of motif in amino acids (AA). Names to the left indicate the clades to which the sequences belong to according to Figures 1-3.


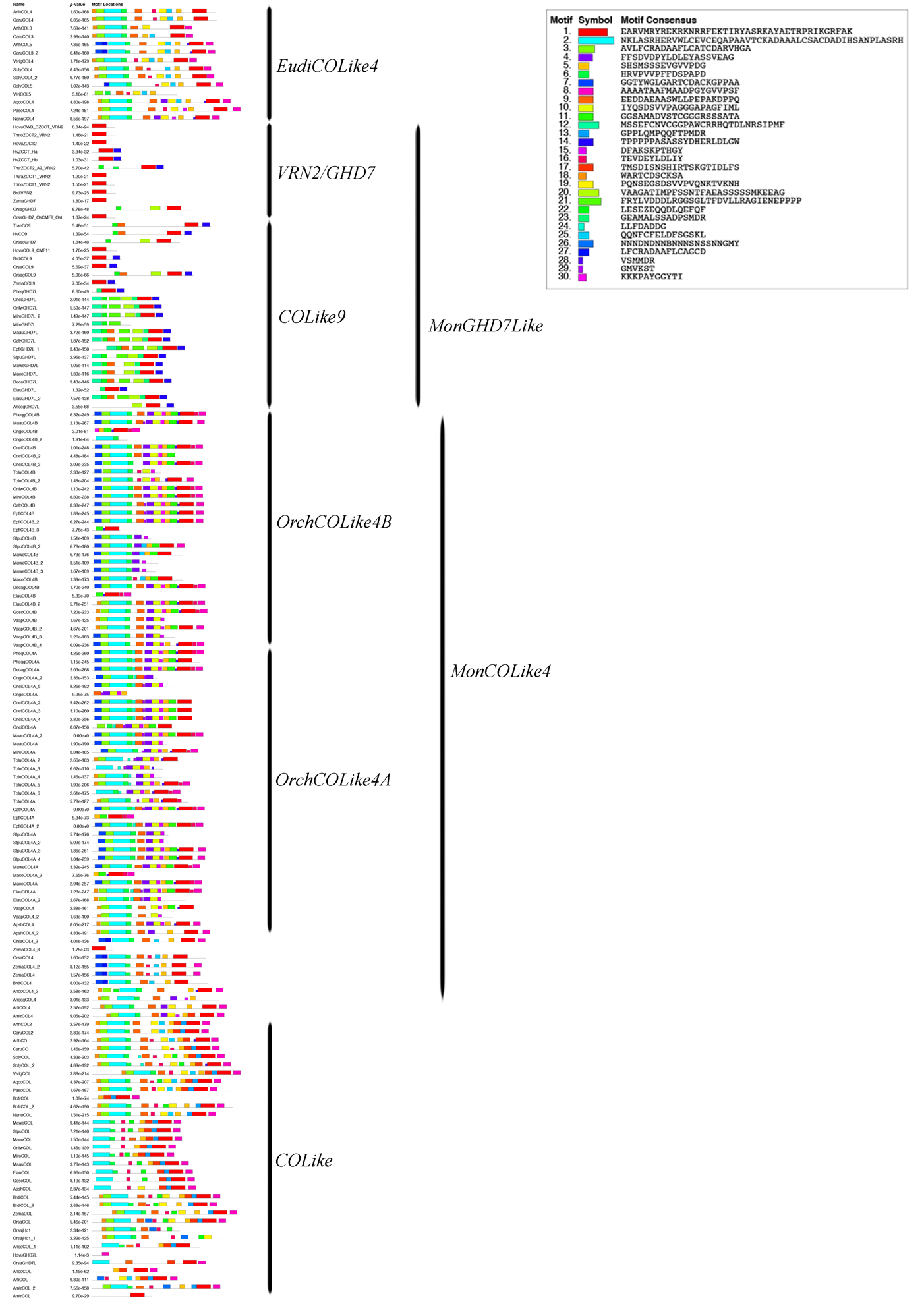


| **Gene Code** | **Sequence** | **Tm (°C)** |
| --- | --- | --- |
| ElauFDL2B_R | AGCGGATGAGGTTCTTTGAA | 56.4 |
| ElauFDL2B_F | CCACCGTGCTTAGCCTTAGT | 60.5 |
| ElauFD2A_R | ATAGTGGTGATCGCCTCCTG | 60.5 |
| ElauFD2A_F | CCCCAAACACCTAAGCGTAA | 58.4 |
| ElauFUL1B_R | GAGTTGGGTCAGGGTCTGAA | 60.5 |
| ElauFUL1B_F | CTGAAGGCAAGGGTTGAGAG | 60.5 |
| ElauFUL1A_R | GTTGTAGTTCTTTGATGCTC | 54.3 |
| ElauFUL1A_F | GCAGTTAAAGCGGATCGAGA | 58.4 |
| ElauCOL4B_2_R | GAACGCTGGCTTGGATTTAG | 58.4 |
| ElauCOL4B_2_F | GTCCCCTTCTTCGATTCTCC | 60.5 |
| ElauCOL4A_R | TTCTCAAACCGCCTGTTCTT | 56.4 |
| ElauCOL4A_2_R | TCCGATGACGACATCTACGA | 58.4 |
| ElauCOL4A_2_ElauCOL4A_F | CTCCCTCAAGGGATGATGAA | 58.4 |
| ElauGHD7L_2_R | CCCTTAATCCTTGGCCTCAT | 58.4 |
| ElauGHD7L_2_F | CGTTCGGGTACTTGGATGAT | 58.4 |
| ElauCOL_R | CATGGGAGTGAACTGGTGTG | 60.5 |
| ElauCOL_F | ACGAGGAAGAGGATGGGTTC | 60.5 |
| ElauSOC1L2_F | GGAAAGACGGAGATGAGAC | 57.3 |
| ElauSOC1L2_R | CTTATGCTGATGATTGTCATC | 55.5 |
| ElauSOC1L1b_F | GAAGGACGGAGATGAGACG | 59.5 |
| ElauSOC1L1b_R | CAGTTCGGTCTCTACATCCT | 58.4 |
| ElauSOC1L1a_F | CGGAGATGAAGCGTATAGAA | 56.4 |
| ElauSOC1L1a_R | CATCCTTATAGTGGCTATCA | 54.3 |
| ACTIN7a_F | GCATTGTGCTTGATTCCGGTGATGGTGT | 55 |
| ACTIN7a_R | CCACCTTAATCTTCATGCTGC | 55 |

Table S2. Primers used for gene expression analysis of *COL/COL4, FD, FLC/FUL* and *SOC1* in orchids. F indicates 5'-3 'primers. R indicates 3'-5 'primers.

| Stat | ElauIMR1 | ElauIMR2 | ElauIMR3 | ElauSAMR1 | ElauSAMR2 | ElauSAMR3 | Elau combined |
| --- | --- | --- | --- | --- | --- | --- | --- |
| Total length of sequence: | 152966663 bp | 175912664 bp | 142320963 bp | 225643328 bp | 223898692 bp | 213165438 bp | 361649590 bp |
| Total number of sequences: | 162650 | 190941 | 149109 | 251236 | 245014 | 235490 | 404381 |
| Average contig length is: | 940 bp | 921 bp | 954 bp | 898 bp | 913 bp | 905 bp | 894 bp |
| Largest contig: | 15129 bp | 19213 bp | 13948 bp | 16835 bp | 16715 bp | 16721 bp | 17049 bp |
| Shortest contig: | 184 bp | 178 bp | 189 bp | 182 bp | 190 bp | 180 bp | 182 bp |
| N25 stats: | 25% of total sequence length is contained in the 9805 sequences >= 2787 bp | 25% of total sequence length is contained in the 10813 sequences >= 2870 bp | 25% of total sequence length is contained in the 9784 sequences >= 2649 bp | 25% of total sequence length is contained in the 13294 sequences >= 2909 bp | 25% of total sequence length is contained in the 13007 sequences >= 2962 bp | 25% of total sequence length is contained in the 12543 sequences >= 2939 bp | 25% of total sequence length is contained in the 20484 sequences >= 3037 bp |
| N50 stats: | 50% of total sequence length is contained in the 27677 sequences >= 1679 bp | 50% of total sequence length is contained in the 30980 sequences >= 1696 bp | 50% of total sequence length is contained in the 26812 sequences >= 1674 bp | 50% of total sequence length is contained in the 39436 sequences >= 1628 bp | 50% of total sequence length is contained in the 38517 sequences >= 1663 bp | 50% of total sequence length is contained in the 37031 sequences >= 1647 bp | 50% of total sequence length is contained in the 61167 sequences >= 1650 bp |
| N75 stats: | 75% of total sequence length is contained in the 60668 sequences >= 757 bp | 75% of total sequence length is contained in the 69682 sequences >= 721 bp | 75% of total sequence length is contained in the 56285 sequences >= 816 bp | 75% of total sequence length is contained in the 92898 sequences >= 665 bp | 75% of total sequence length is contained in the 90531 sequences >= 679 bp | 75% of total sequence length is contained in the 86815 sequences >= 673 bp | 75% of total sequence length is contained in the 148607 sequences >= 643 bp |
| Total GC count: | 63101114 bp | 71460266 bp | 59378562 bp | 90611988 bp | 90136612 bp | 85896589 bp | 142800441 bp |
| GC %: | 41.25% | 40.62% | 41.72% | 40.16% | 40.26% | 40.30% | 39.49% |
| Number of Ns: | 0 | 0 | 0 | 0 | 0 | 0 | 0 |
| Ns %: | 0.00% | 0.00% | 0.00% | 0.00% | 0.00% | 0.00% | 0.00% |

Table S3. *De novo* assembly of transcriptomes of *Elleanthus aurantiacus* of the vegetative (SAM) and reproductive (IM) meristems.

| Model | Gene clade | Results | InL | 2ΔInL | LRT | Evolution rate |
| --- | --- | --- | --- | --- | --- | --- |
| 1-ω |  | ω_0_ = 0.0519 | -12.423,15 | - | - | Purifying selection |
| 2-ω | *COL* | ωb = 0.1792 | -12.236,20 | 23,22 | P < 0.001 | Increased purifying selection |
|  |  | ω_f_ = 0.0580 |  |  |  |  |
|  | *MonGHD7L* | ωb = 0.1680 | -12.246,54 | 3,63 | P < 0.5 | Relaxed purifying selection |
|  |  | ω_f_ = 0.2832 |  |  |  |  |
|  | *MonCOL4* | ωb = 0.1692 | -12.245,52 | 5.53 | P < 0.05 | Relaxed purifying selection |
|  |  | ω_f_ = 0.4761 |  |  |  |  |
| 1-ω |  | ω_0_ = 0.0332 | -4.533,45 | - | - | Purifying selection |
| 2-ω | *MonFDL1* | ωb = 0.0502 | -4.525,08 | 10,72 | P < 0.001 | Increased purifying selection |
|  |  | ω_f_ = 0.0053 |  |  |  |  |
|  | *OrchFDL1* | ωb = 0.0503 | -4.35,00 | 10,22 | P < 0.001 | Relaxed purifying selection |
|  |  | ω_f_ = 0.1235 |  |  |  |  |
|  | *OrchFDL2A* | ωb = 0.0527 | -4.526,11 | 12,57 | P < 0.001 | Relaxed purifying selection |
|  |  | ω_f_ = 0.1553 |  |  |  |  |
|  | *OrchFDL2B* | ωb = 0.0421 | -4.528,91 | 8,93 | P = 0.01 | Relaxed purifying selection |
|  |  | ω_f_ = 0.0799 |  |  |  |  |
| 1-ω |  | ω_0_ = 0.4510 | -11.193,91 | - | - | Purifying selection |
| 2-ω | *EudiFLC* | ωb = 0.5102 | -11.192,03 | 5,62 | P < 0.05 | Increased purifying selection |
|  |  | ω_f_ = 0.4086 |  |  |  |  |
|  | *MonFLC* | ωb = 0.4344 | -11.191,50 | 6,63 | P < 0.05 | Relaxed purifying selection |
|  |  | ω_f_ = 0.6191 |  |  |  |  |
|  | *EudiAP1/FUL* | ωb = 0.4752 | -11.193.01 | 3,67 | P < 0.5 | Increased purifying selection |
|  |  | ω_f_ = 0.3761 |  |  |  |  |
|  | *VRN1* | ωb = 0.3742 | -11.192,96 | 3,74 | P = 0.90 | Relaxed purifying selection |
|  |  | ω_f_ = 0.4755 |  |  |  |  |
|  | *MonFUL1* | ωb = 0.4390 | -11.189,20 | 11,24 | P < 0.001 | Relaxed purifying selection |
|  |  | ω_f_ = 0.9332 |  |  |  |  |
|  | *MonFUL2* | ωb = 0.3971 | -11.185,08 | 19,50 | P < 0.001 | Relaxed purifying selection |
|  |  | ω_f_ = 0.6250 |  |  |  |  |
| 1-ω |  | ω_0_ = 0.2266 | -29.103,24 | - | - | Purifying selection |
| 2-ω | *EudiAGL42/71/72* | ωb = 0.2350 | -29.080,40 | 46,89 | P < 0.001 | Increased purifying selection |
|  |  | ω_f_ = 0.1386 |  |  |  |  |
|  | *EudiAGL14/19* | ωb = 0.2213 | -29.095,31 | 17,88 | P < 0.001 | Increased purifying selection |
|  |  | ω_f_ = 0.1260 |  |  |  |  |
|  | *EudiSOC1/AGL20* | ωb = 0.2476 | -29.090,43 | 27,84 | P < 0.001 | Increased purifying selection |
|  |  | ω_f_ = 0.1844 |  |  |  |  |
|  | *OrchSOC1L 1A* | ωb = 0.2166 | -29.103,49 | 1,56 | P = 0.9 | Increased purifying selection |
|  |  | ω_f_ = 0.1672 |  |  |  |  |
|  | *OrchSOC1L 1B* | ωb = 0.2197 | -29.103,30 | 1,78 | P= 0.95 | Increased purifying selection |
|  |  | ω_f_ = 0.1967 |  |  |  |  |
|  | *OrchSOC1L 2* | ωb = 0.2270 | -29.091,00 | 25,90 | P < 0.001 | Increased purifying selection |
|  |  | ω_f_ = 0.1430 |  |  |  |  |

Table S4. Evolutionary rates of *COL/COL4, FD, FLC/FUL* and *SOC1* gene clades that are evolving at statistically different rates. Comparison of the one ratio model (1-ω) assumes a constant dN/dS ratio ( = ω, per site ratio of nonsynonymous -dN- to synonymous -dS- substitution) along tree branches, against a two-ratio model (2-ω) that assumes a different ratio for a designated subclade (foreground -ωf) relative to the remaining sequences (background -ωb). For each of the LRTs, twice the difference of log likelihood between the models (2ΔInL) was compared to critical values from a χ^2^ distribution, with degree of freedom equal to the differences in number of estimated parameters between models. The test was conducted for the domains B-box I, B-box II, and a CCT for *COL/COL4* genes; bZIP and SAP domains for FD genes; MADS, I and K for *FLC/FUL* genes; and complete MIKC domains for *SOC1*.

**Figure S2.**  Conserved motifs of FD proteins across flowering plants identified with a MEME analysis. Each motif is represented by a coloured box with the corresponding summarized sequence at the right. Size of bar indicates size of motif in amino acids (AA). Names to the left indicate the clades to which the sequences belong to according to Figure 4. Green box on the right indicates motif 6 correspond to the SAP motif at C-terminal end with the putative phosphorylation site (S164) marked with a black arrow.

**Figure S3.** Conserved motifs of FLC/FUL proteins across flowering plants identified with a MEME analysis. Each motif is represented by a coloured box with the corresponding summarized sequence at the right. Size of bar indicates size of motif in amino acids (AA). Names to the left indicate the clades to which the sequences belong to according to Figure 5.


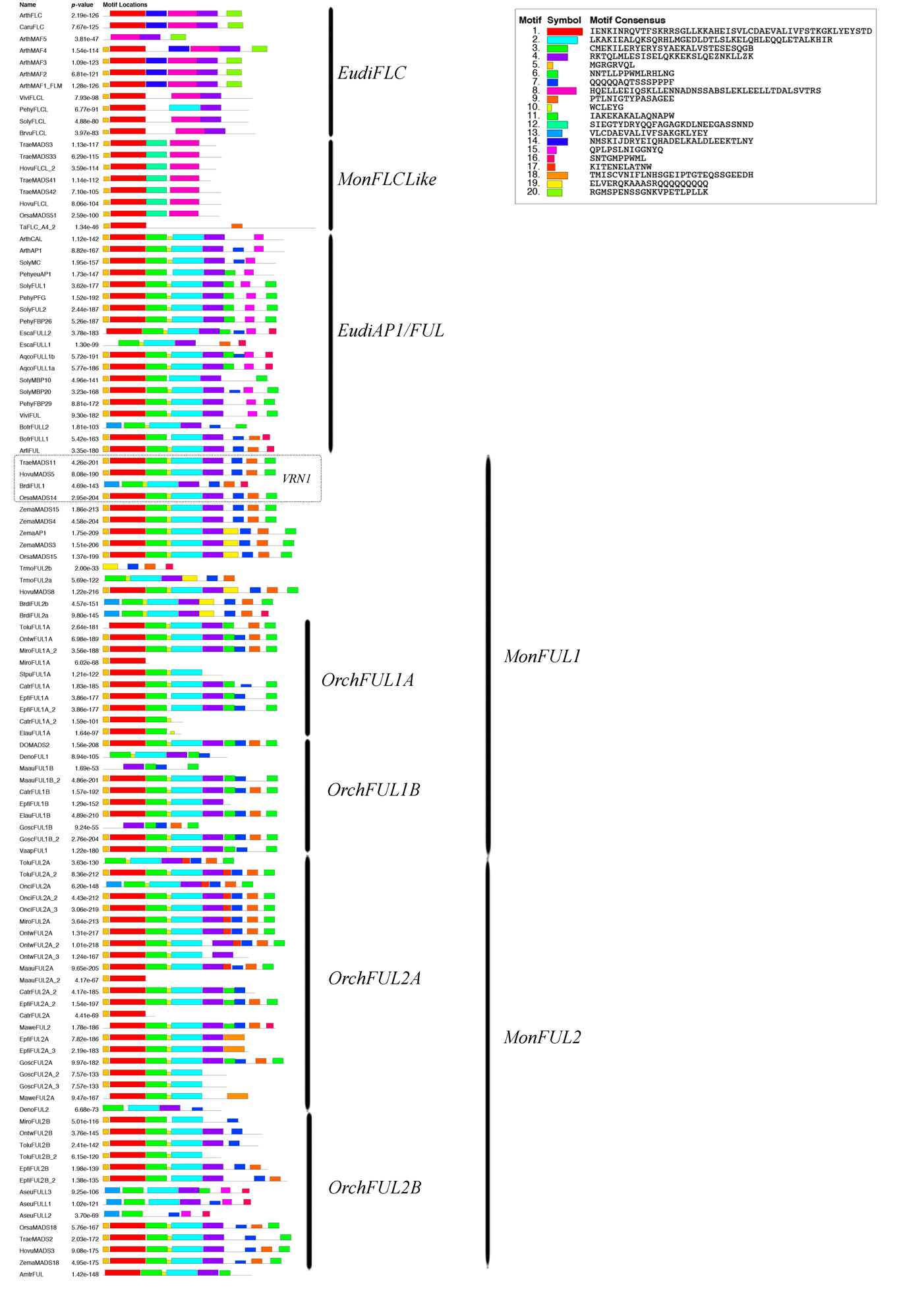


**Figure S4.**  Conserved motifs of SOC1 proteins across flowering plants identified with a MEME analysis. Each motif is represented by a coloured box with the corresponding summarized sequence at the right. Size of bar indicates size of motif in amino acids (AA). Names to the left indicate the clades to which the sequences belong to according to Figure 6.


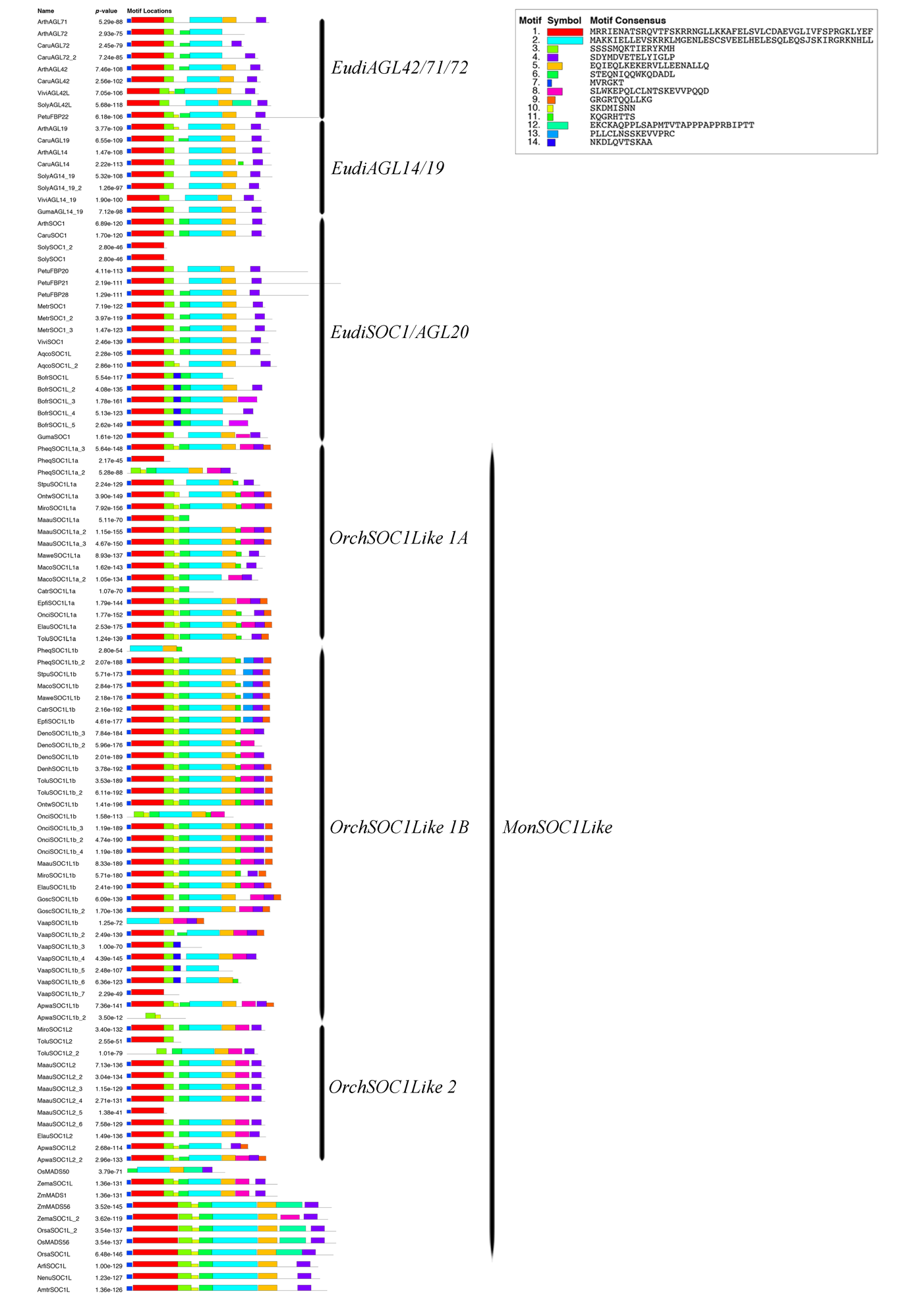


**Figure S5.** Expression patterns of selected *COL/COL4* homologs from model species *Arabidopsis thaliana, Oryza sativa* and *Zea Mays*. Only gene expression patterns available are shown. Names inside parenthesis correspond to clades according to Figures 1-3. Taken from the BAR (http://bar.utoronto.ca/).


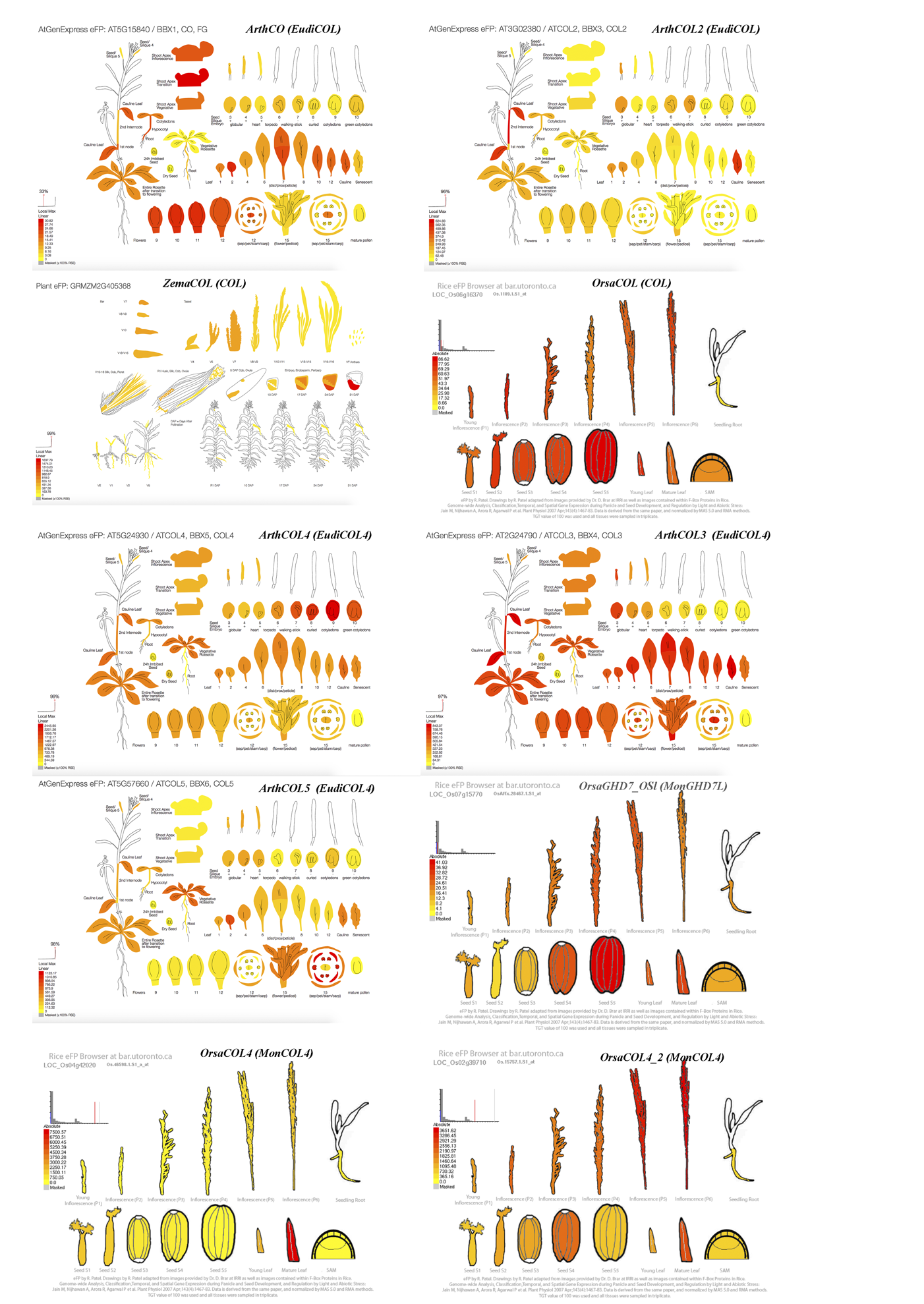

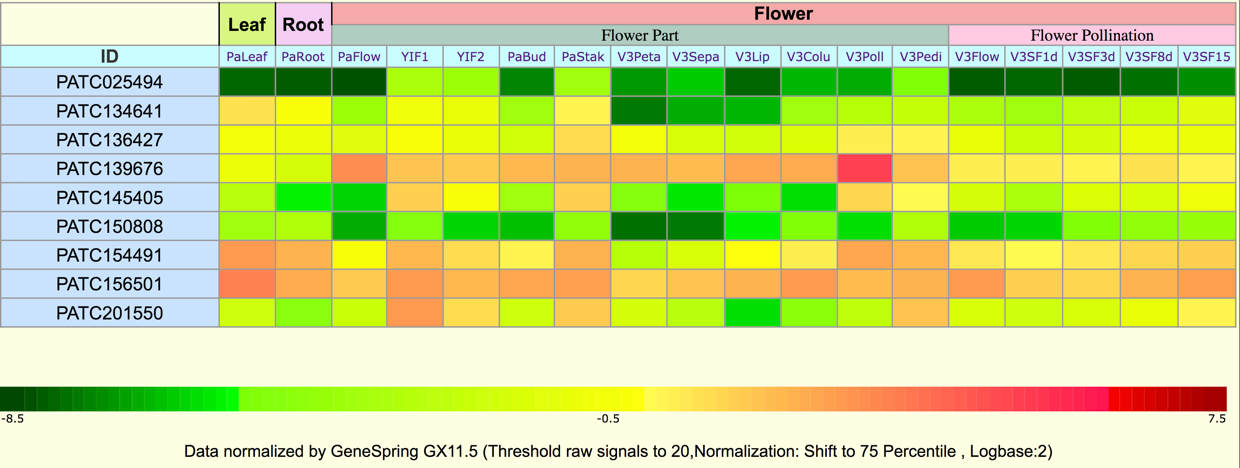


Table S5. Expression levels of *COL/COL4, FD, FLC/FUL* and *SOC1* by Microarrays in *Phalaenopsis aphrodite* genome. Expression colors follow the bar of the bottom. ID genes correspond to: PATC025494: *PhapFDL2B*, PATC134641: *PhapFDL2A*, PATC139676: *PhapCOL4A*, PATC156501: *PhapCOL4B*, PATC145405: *PhapFUL1*, PATC201550: *PhapFUL2*, PATC136427: *PhapSOC1L1b*, PATC150808: *PhapSOC1L1b*_2, PATC154491: *PhapSOC1L1a*. The dissections correspond to genomic/transcriptomic information available in the database, from left to right: PaLeaf: Mature leaf, PaRoot: Root tip 1cm, PaFlow: Full blossom flower, YIF1: Young inflorescence, YIF2: Inflorescence, PaBud: Flower bud, PaStak: Flower stalk, V3Peta: Petal, V3Sepa: Sepal, V3Lip: Labellum, V3Colu: Gynostemium, V3Poll: Pollinia, V3Pedi: Pedicel, V3Flow: Full blossom flower, V3SF1d: Senescence flower 1DAP, V3SF3d: Senescence flower 3DAP, V3SF8d: Senescence flower 8DAP, V3SF15: Senescence flower 15DAP. Data available on Orchidstra 2.0: <http://orchidstra2.abrc.sinica.edu.tw/orchidstra2/index.php>

| **Gene** | **Gene Id** | **Se** | **Pe** | **La** | **Po** | **Gy** | **FS** | **L** | **R** | **4S** | **7S** | **12S** |
| --- | --- | --- | --- | --- | --- | --- | --- | --- | --- | --- | --- | --- |
| *PheqFDL2B* | Peq000940 | 0 | 0 | 0.45 | 0.24 | 0.31 | 0 | 0 | 1.83 | 0 | 0 | 0.31 |
| *PheqFDL2A* | Peq016774 | 0 | 0.89 | 0.74 | 1.56 | 1.47 | 0 | 0 | 0 | 2.28 | 6.41 | 5.73 |
| *PheqgCOL4A* | Peq002718 | 184.97 | 157.94 | 124.98 | 44.34 | 107.66 | 154.36 | 0 | 472.85 | 73.25 | 61.1 | 106.66 |
| *PheqgCOL4B* | Peq004392 | 59.82 | 83.19 | 82.53 | 36.27 | 35.6 | 72.57 | 889.21 | 166.04 | 29.88 | 32.14 | 56.92 |
| *PheqFUL2* | Peq013900 | 2.98 | 8.77 | 9.08 | 8.9 | 18.08 | 0 | 0 | 21.58 | 3.26 | 11.8 | 15.2 |
| *PheqFUL1* | Peq021805 | 0.87 | 1.91 | 1.66 | 0.87 | 9.35 | 0 | 0 | 0 | 0 | 0.38 | 0.07 |
| *PheqSOC1L1b_2* | Peq009223 | 12.49 | 9.87 | 13.19 | 12.09 | 16.78 | 0 | 0 | 41.8 | 0 | 0 | 0.08 |
| *PheqSOC1L1a_3* | Peq000726 | 15.94 | 30.28 | 40.75 | 169.76 | 53.96 | 0 | 0 | 31.91 | 0.4 | 0.08 | 0.39 |

Table S6. Expression levels of *COL/COL4, FD, FLC/FUL* and *SOC1* by FPKM in *Phalaenopsis equestris* genome. The dissections correspond to genomic/transcriptomic information available in the database, from left to right: Se: Sepal, Pe: Petal, La: Labellum, Po: Pollinium, Gy: Gynostemium, FS: Floral stalk, L: Leaf, R: Root, 4S: 4 Day Seed, 7S: 7 Day Seed, 12S: 12 Day Seed. Data available on OrchidBase 4.0: <http://orchidbase.itps.ncku.edu.tw/est/home2012.aspx>

| **Gene** | **Gene id** | **FB** | **Se** | **La** | **Po** | **Gy** | **St** | **L** | **R** | **GR** | **WR** |
| --- | --- | --- | --- | --- | --- | --- | --- | --- | --- | --- | --- |
| *DecaFDL2A_3* | Dca016995 | 0 | 0 | 0 | 0.16 | 0 | 5.79 | 20.74 | 24.29 | 27.96 | 9.05 |
| *DecaFDL2B_2* | Dca022842 | 0.07 | 0 | 0 | 0.05 | 0.14 | 9.55 | 0.59 | 3.59 | 1.1 | 1.93 |
| *DecagCOL4A* | Dca008867 | 146.25 | 64.29 | 81.63 | 216.48 | 94.23 | 293.62 | 426.35 | 75.36 | 111.85 | 129.51 |
| *DecagCOL4B* | Dca023344 | 125.57 | 394.94 | 420.11 | 60.4 | 168 | 271.85 | 506.61 | 59.59 | 185.58 | 65.42 |
| *DecaFUL2* | Dca016066 | 66.62 | 7.1 | 61.55 | 35.64 | 74.29 | 325.05 | 185.72 | 107.97 | 96.52 | 74 |
| *DecaFUL1* | Dca013065 | 26.64 | 17.83 | 5.56 | 39.93 | 37.08 | 66.75 | 11.27 | 65.25 | 68.78 | 40.59 |
| *DecaSOC1L2* | Dca013492 | 55.45 | 69.29 | 138.95 | 6.56 | 98.24 | 16.61 | 13.59 | 82.2 | 49.89 | 44.37 |
| *DecaSOC1L1a* | Dca021303 | 22 | 29.89 | 22.86 | 126.78 | 31.33 | 43.72 | 25.91 | 45.29 | 24.74 | 16.43 |
| *DecaSOC1L1b* | Dca001311 | 38.23 | 67.31 | 77.95 | 64.84 | 76.65 | 151.54 | 111.99 | 119.2 | 125.29 | 101.12 |

Table S7. Expression levels of *COL/COL4, FD, FLC/FUL* and *SOC1* by FPKM in *Dendrobium catenatum* genome. The dissections correspond to genomic/transcriptomic information available in the database, from left to right: FB: Flower bud, Se: Sepal, La: Labellum, Po: Pollinium, Gy: Gynostemium, St: Stem, L: Leaf, R: Root, GR: Green Root Tip, WR: White Part Root. Data available on OrchidBase 4.0: <http://orchidbase.itps.ncku.edu.tw/est/home2012.aspx>

| **Gene** | **Gene id** | **I** | **L** | **R** | **S** | **St** | **T** | **Po** |
| --- | --- | --- | --- | --- | --- | --- | --- | --- |
| *ApshFDL1* | Ash014780 | 10.39 | 9.59 | 2.54 | 21.4 | 8.41 | 83.58 | 2.31 |
| *ApshCOL* | Ash001160 | 34.9 | 96.97 | 7.96 | 16.04 | 25.21 | 20.51 | 29.94 |
| *ApshCOL4_2* | Ash015904 | 21.18 | 27.72 | 2.77 | 138.56 | 128.77 | 7.57 | 25.36 |
| *ApshCOL4* | Ash005534 | 73.32 | 322.26 | 24.28 | 42.64 | 35.38 | 5.14 | 31.57 |
| *ApshFUL2* | Ash012887 | 41.19 | 128.02 | 22.4 | 77.92 | 32.76 | 6.32 | 52.17 |
| *ApshFUL1* | Ash002327 | 127.73 | 1550.58 | 92.59 | 290.52 | 482.67 | 115.76 | 7.31 |
| *ApshSOC1L1* | Ash007343 | 4 | 2.62 | 4.39 | 43.99 | 9.63 | 17.85 | 9.08 |

Table S8. Expression levels of *COL/COL4, FD, FLC/FUL* and *SOC1* by FPKM in *Apostasia schenzhenica* genome. The dissections correspond to genomic/transcriptomic information available in the database, from left to right: I: Inflorescence, L: Leaf, R: Root, S: Seed, St: Stem, T: Tuber, Po: Pollinium. Data available on OrchidBase 4.0: <http://orchidbase.itps.ncku.edu.tw/est/home2012.aspx>

**Figure S6.** Expression patterns of selected *FD* homologs from model species *Arabidopsis thaliana, Oryza sativa* and *Zea Mays*. Only gene expression patterns available are shown. Names inside parenthesis correspond to clades according to Figure 4. Taken from the BAR (http://bar.utoronto.ca/).

S
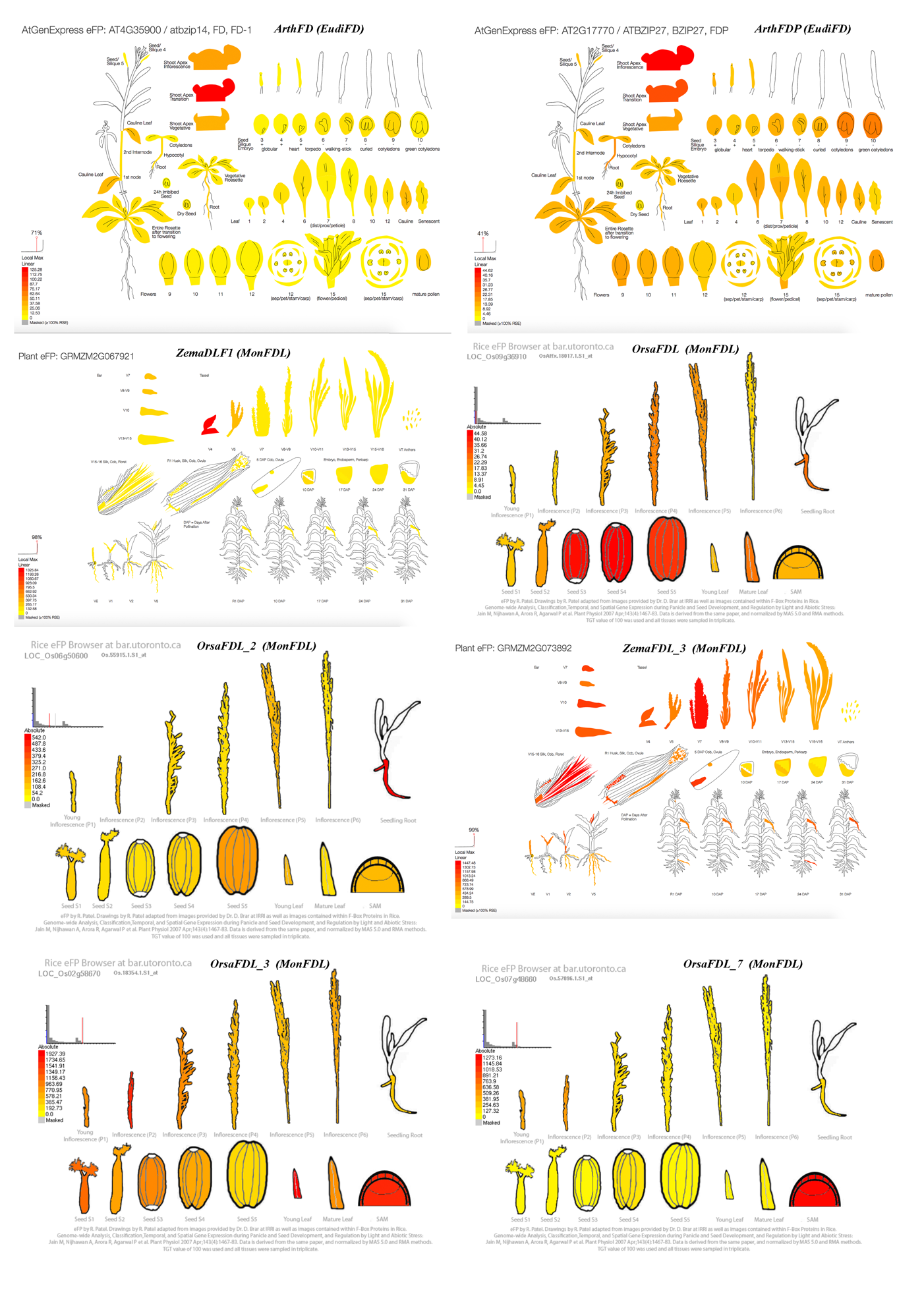


**Figure S7.** Expression patterns of *EudiFLC* homologs from model species *Arabidopsis thaliana*. Only gene expression patterns available are shown. Taken from the BAR (http://bar.utoronto.ca/).


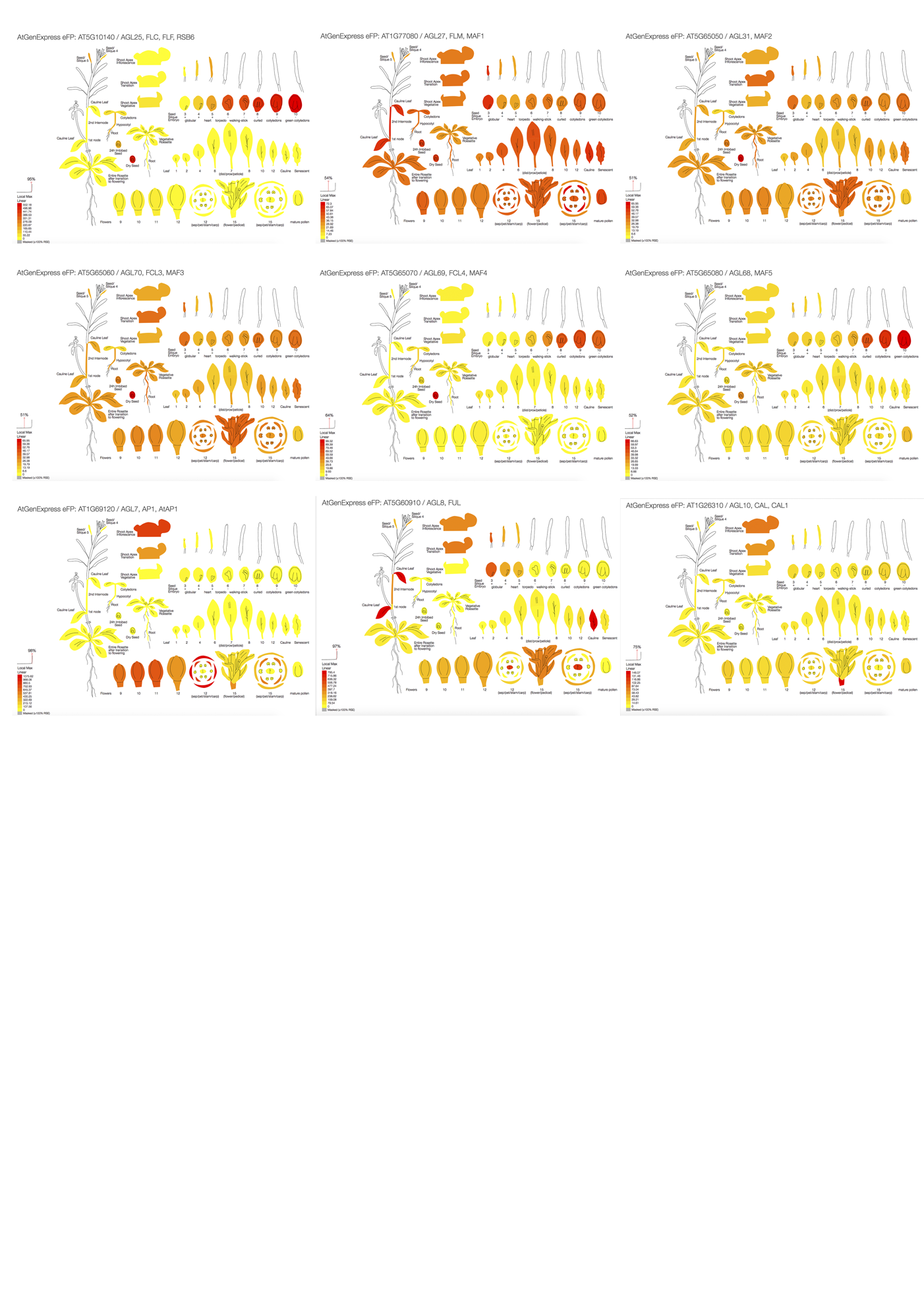


**Figure S8.** Expression patterns of *FUL* homologs from model species*, Oryza sativa* and *Zea Mays* and *FLC* homologs from *Tritricum aestivum*. Only gene expression patterns available are shown. Names inside parenthesis correspond to clades according to Figure 5. Taken from the BAR (http://bar.utoronto.ca/).


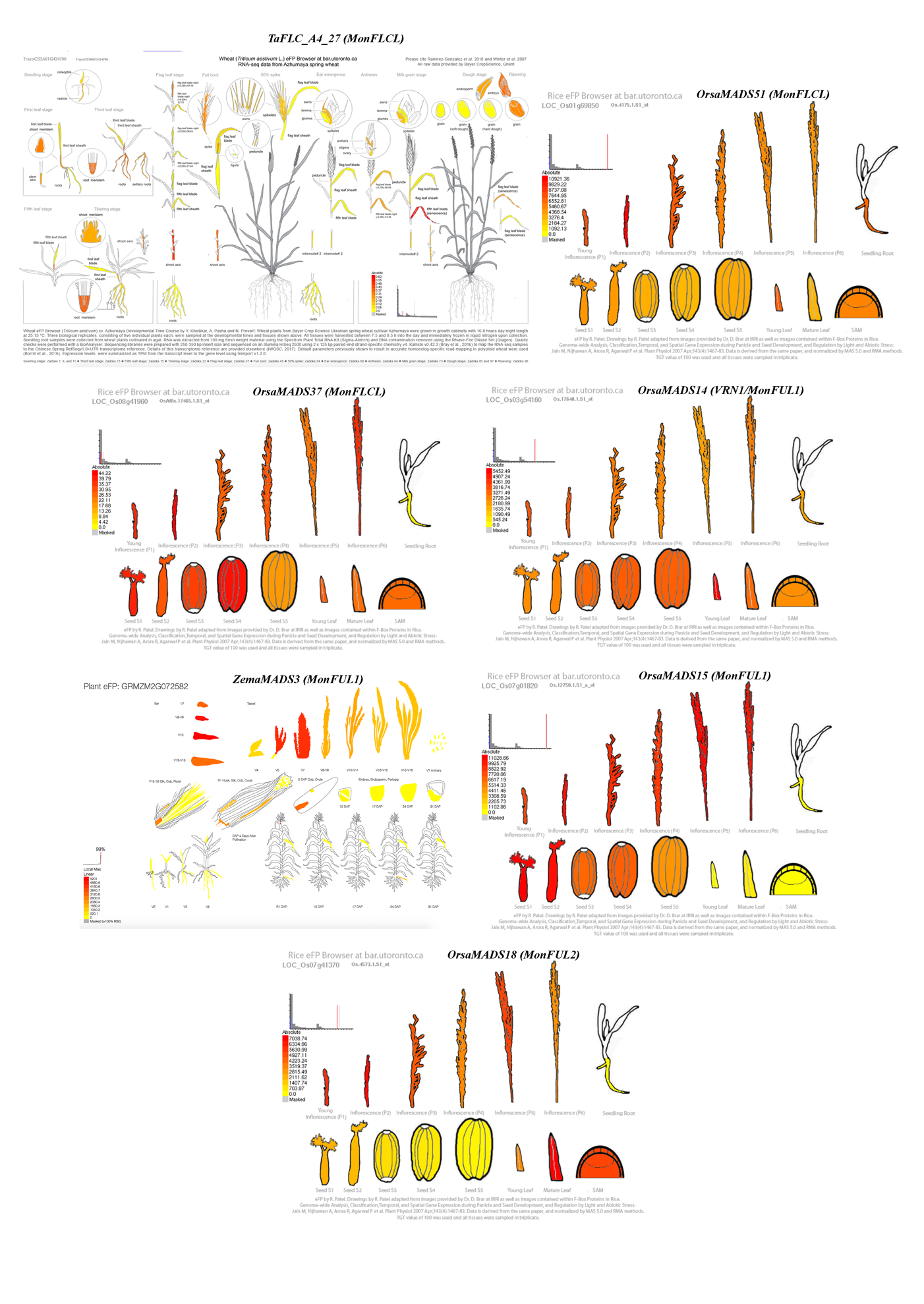


**Figure S9.** Expression patterns of selected *SOC1* homologs from model species *Arabidopsis thaliana, Oryza sativa* and *Zea Mays.* Only gene expression patterns available are shown. Names inside parenthesis correspond to clades according to Figure 6. Taken from the BAR (http://bar.utoronto.ca/).


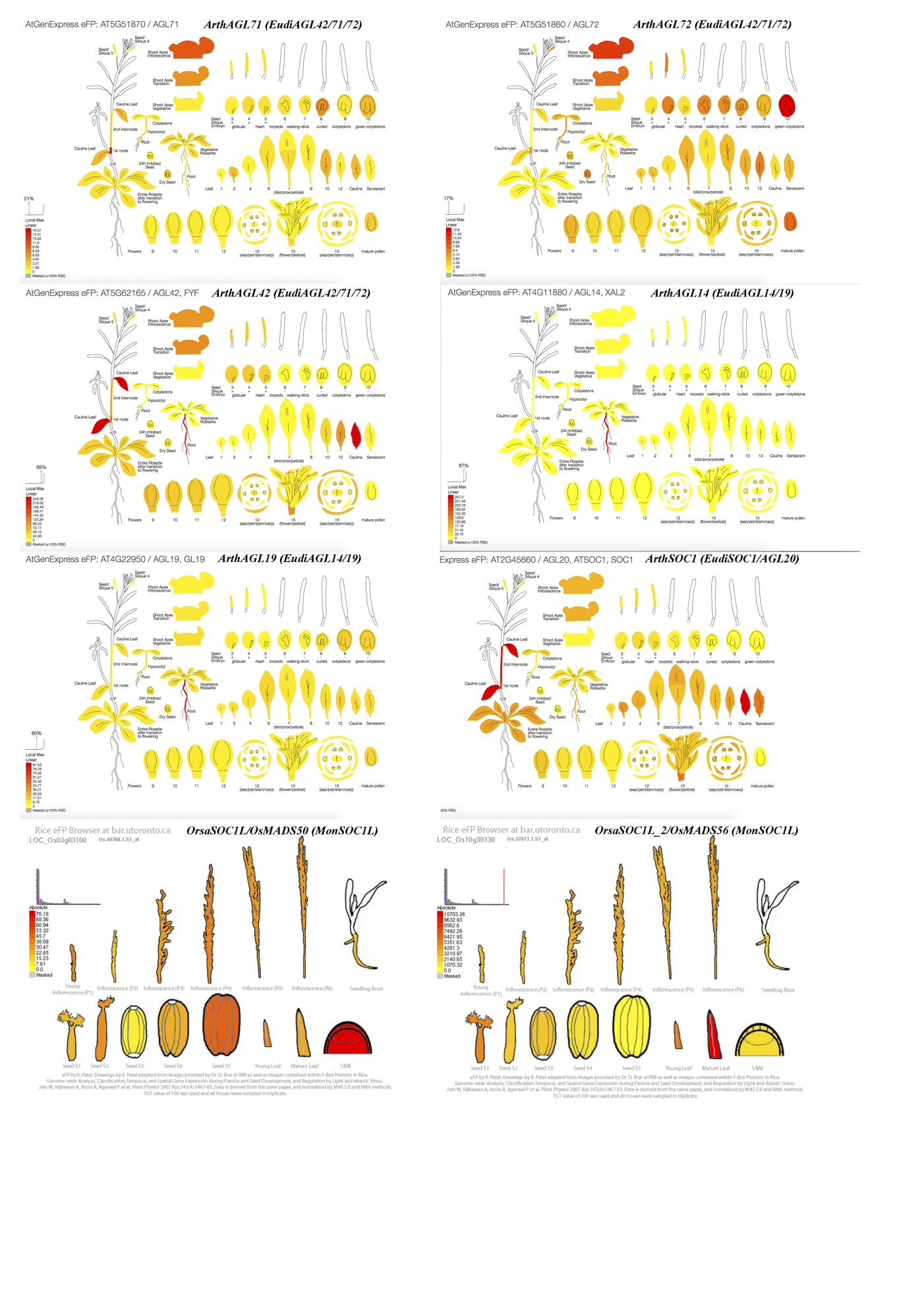

Supplement: Supplementary file 1 — Supplementary file1 (DOCX 74830 KB) [file 497_2023_482_MOESM1_ESM.docx]
